# Interactions with alloparents are associated with the diversity of infant skin and fecal bacterial communities in Chicago, United States

**DOI:** 10.1002/ajhb.23972

**Published:** 2023-08-26

**Authors:** Melissa B. Manus, Maria Luisa Savo Sardaro, Omolola Dada, Maya I. Davis, Melissa R. Romoff, Stephanie G. Torello, Esther Ubadigbo, Rebecca C. Wu, Emily S. Miller, Katherine R. Amato

**Affiliations:** ^1^ Department of Anthropology Northwestern University Evanston Illinois USA; ^2^ Department of Human Science and Promotion of the Quality of Life University of San Raffaele Rome Italy; ^3^ Department of Obstetrics and Gynecology, Division of Maternal Fetal Medicine Feinberg School of Medicine, Northwestern University Chicago Illinois USA

## Abstract

Introduction: Social interactions shape the infant microbiome by providing opportunities for caregivers to spread bacteria through physical contact. With most research focused on the impact of maternal–infant contact on the infant gut microbiome, it is unclear how alloparents (i.e., caregivers other than the parents) influence the bacterial communities of infant body sites that are frequently contacted during bouts of caregiving, including the skin.

Methods: To begin to understand how allocare may influence the diversity of the infant microbiome, detailed questionnaire data on infant–alloparent relationships and specific allocare behaviors were coupled with skin and fecal microbiome samples (four body sites) from 48 infants living in Chicago, United States.

Results: Data from 16S *rRNA* gene amplicon sequencing indicated that infant skin and fecal bacterial diversity showed strong associations (positive and negative) to having female adult alloparents. Alloparental feeding and co‐sleeping displayed stronger associations to infant bacterial diversity compared to playing or holding. The associations with allocare behaviors differed in magnitude and direction across infant body sites. Bacterial relative abundances varied by infant–alloparent relationship and breastfeeding status.

Conclusion: This study provides some of the first evidence of an association between allocare and infant skin and fecal bacterial diversity. The results suggest that infants' exposure to bacteria from the social environment may vary based on infant–alloparent relationships and allocare behaviors. Since the microbiome influences immune system development, variation in allocare that impacts the diversity of infant bacterial communities may be an underexplored dimension of the social determinants of health in early life.

## 1 INTRODUCTION

Early life environments influence infant biology and health through multiple routes, including by exposing infants to bacteria that are critical for nutrition, immune function, and other aspects of physiology (Ahmed et al., 2014; Dowd & Renson, 2018; Gensollen et al., 2016; Yang et al., 2016). Many environmentally sourced bacteria colonize the infant body and contribute to the development of the microbiome―the collection of bacteria (and their genes) found in and on various body sites, including the gut, skin, and oral cavity―which influences both short and long‐term health. In early life, the social environment facilitates bacterial acquisition, as newborns initially receive a considerable dose of bacteria from their mothers' bodies during delivery, the first few months of breastfeeding, and skin‐to‐skin contact (Asnicar et al., 2017; Fehr et al., 2020; Ferretti et al., 2018; Korpela et al., 2018). In addition, there is evidence that the infant gut is colonized by bacteria that stem from fathers'/co‐parents' bodies (Korpela et al., 2018), and may also be affected by the presence of siblings who contribute bacteria to the shared household environment (Azad et al., 2013; Hasegawa et al., 2017; Lane et al., 2019; Laursen et al., 2017).

The small number of studies that consider infants' social environments (Azad et al., 2013; Hasegawa et al., 2017; Korpela et al., 2018; Lane et al., 2019; Laursen et al., 2015; Manus et al., 2020, 2023) focus on variables related to the presence or number of individuals within infants' households or caregiving networks, but do not include data on the specific behaviors that put infants into contact with other people. As a result, our understanding of potential routes of bacterial sharing is limited to assumptions about the types of social interactions that infants may have with different caregivers. For example, a study of infants living in five countries across Europe and North America showed that the infant gut microbiome harbors bacteria that originate from fathers' bodies (Korpela et al., 2018), yet lacked data on the frequency or types of father‐infant interactions that could facilitate bacterial transmission. Similarly, previous studies that tested the association between having older siblings and the composition of the infant gut microbiome did not include information on the specific behaviors that promote physical contact between infants and siblings (Azad et al., 2013; Hasegawa et al., 2017; Lane et al., 2019; Laursen et al., 2017). Without behavioral data, the relative contributions of different individuals (e.g., grandmothers vs. siblings) and types of caregiving behaviors (e.g., feeding vs. holding) to the diversity and development of infant bacterial communities remain poorly understood.

An ecological perspective (Manus, 2021) helps narrow the scope of early life social environments and highlights bouts of caregiving as potential opportunities for the dispersal of bacteria to infants. Here, the bacterial communities of frequently contacted infant body sites, such as the hand and cheek, may experience increased rates of bacterial dispersal during physical contact with caregivers (e.g., during holding, playing, or kissing). In contrast, while caregivers may have less frequent contact with the infant axilla and gut, these body sites are likely shaped by different types of bacterial exposures (e.g., bacteria from clothing or from toys that are put into the mouth). This pattern is supported by studies of the adult skin microbiome, where cohabiting individuals display greater bacterial similarity between skin sites that come into frequent contact with the surrounding environment (Ross et al., 2017). In addition to inter‐individual bacterial sharing, common “self‐contact behaviors” (Manus, 2021) such as putting body parts (e.g., hands and feet) into the mouth may spread bacteria across the infant skin and potentially to the oral and gut microbiomes (Shaffer & Lozupone, 2018). Since the microbiome trains and regulates the immune system in early life (Baviera et al., 2014; Gensollen et al., 2016; Naik et al., 2012; Wanke et al., 2011), the transfer of bacteria to various infant body sites may represent a link between social behaviors, caregiving networks, and infant health.

Research at the intersection of early life social environments and the microbiome is salient to broader themes in human biology. As cooperative breeders, human mothers rely on assistance from alloparents (i.e., non‐parental caregivers) to help care for dependent offspring (Hrdy, 2009; Kaplan et al., 2000; Kramer, 2010). As a result, human infancy is a highly social period during which infants interact with individuals other than their parents. Based on the propensity for bacteria to disperse between individuals (Asnicar et al., 2017; Ferretti et al., 2018; Yassour et al., 2018), bouts of allocare that involve direct physical contact, such as holding, carrying, and feeding, likely promote bacterial dispersal from alloparents to infants. Given the influence of the microbiome on immune activity in early life (Baviera et al., 2014; Naik et al., 2012; Wanke et al., 2011), alloparents' contributions to the developing infant microbiome may be an understudied benefit of allocare to infant health. Further, variation in infant–alloparent interactions across geographic and cultural settings (Martin et al., 2020; Meehan, 2005) may result in different patterns of bacterial sharing, suggesting an additional mechanism by which early life environments can shape health disparities across infant populations (Amato et al., 2021; Findley et al., 2016; Gensollen et al., 2016; Ishaq et al., 2019; McDade et al., 2013; Stinson, 2019).

To assess the relative contributions of different components of allocare (infant–alloparent relationships and specific allocare behaviors) to the diversity of infant bacterial communities, we coupled detailed questionnaire data with skin and fecal microbiome samples (a proxy of the bacterial community in the gastrointestinal tract) collected from infants living in Chicago, IL, United States. The collection of microbiome samples from three different skin sites was motivated by the overall paucity of data on the development of the infant skin microbiome, as well as the fact that many allocare behaviors involve direct skin‐to‐skin contact that likely creates opportunities for bacterial sharing to the infant skin. Since the sequencing technology used in this study describes the taxonomic composition of bacteria (and not other types of microorganisms) in each sample, we use the term “bacterial” when referring to specific results of the current study and others that used similar sequencing platforms.

This article evaluated a series of hypotheses. First, we expected that infants with more alloparents would display elevated bacterial diversity across multiple body sites (H1), since having a greater number of alloparents may expose infants to a more diverse pool of bacteria available for dispersal. Based on evidence that cultural and gender norms related to infant caregiving can influence who provides allocare (Brito et al., 2019; Martin et al., 2020; Meehan, 2005), we expected to find the strongest associations between infant bacterial diversity and having female adult alloparents, compared to sibling or male adult alloparents (H2). This prediction was built on the assumption that in this study population, female adult alloparents provide more (frequency and/or intensity) allocare than male adult or sibling alloparents. Due to the different frequencies with which infant body sites are contacted during allocare, we also expected to see variation in infant bacterial diversity in relation to specific allocare behaviors (H3) and across infant body sites (H4). Finally, we expected that the relative abundances of “core” bacteria (see Materials and Methods) would vary based on having adult or sibling alloparents, as well as by breastfeeding status (H5). Given the paucity of information about the infant skin microbiome in the existing literature, particularly in relation to allocare, we did not construct directed predictions for hypotheses 3–5.

## 2 MATERIALS AND METHODS

This cross‐sectional study collected 144 skin swab samples and 27 fecal samples from 48 infants whose mothers were recruited from four different locations (Figure S1). Exclusion criteria included current COVID‐19 infection of the infant or any household member; permanent residence outside of Chicago or the immediate suburbs; mothers' need for an English interpreter; current maternal diabetes, hypertension, or cancer; and infant admittance to the NICU. We excluded participants with these documented health conditions based on known correlations to microbiome diversity and composition in both pregnancy and infancy (Hu et al., 2013; Pammi et al., 2017). We did not restrict recruitment based on participants' socioeconomic status, race, or ethnicity. This study was approved by the Institutional Review Board of Northwestern University (study number #STU00210184).

Between April and June of 2021, mothers were recruited on the postpartum floor of Northwestern's Prentice Women's Hospital in Chicago. Eligible mothers were contacted in‐person by MBM and introduced to the study. Informed consent was obtained from interested mothers on behalf of her infant at the time of recruitment. Mothers received materials for at‐home infant fecal sample collection (gloves and collection vial) to be completed ~6 weeks after enrollment. MBM contacted mothers by email at 5 weeks postpartum to arrange a time for collecting infant skin swab samples and obtaining the fecal sample. Due to logistical constraints, one infant was sampled at 4 weeks, while another was sampled at 6 months. Skin swab samples were collected either at a park in Chicago or at participants' homes (often outdoors). At this time, mothers returned the infant fecal sample, which had been stored in a home freezer (and on ice packs during transport) for no more than 1 day.

Between July and October of 2021, mothers with infants under 6 months of age were recruited via email, fliers, and word‐of‐mouth through a family practice clinic in a Western suburb of Chicago, a local doula practice, and existing research registries at Northwestern University. Informed consent took place at the time of sample collection for all recruitment locations outside of the hospital, and fecal samples were collected opportunistically at the time of skin swabbing (i.e., if the infant produced a soiled diaper during the study visit). At the clinic, physicians and/or medical staff introduced the project by phone or in‐person to mothers who were visiting the clinic for routine postnatal appointments. MBM provided interested mothers with additional study information and collected samples from infants during the appointment. Additionally, mothers were recruited through the local doula practice and university research registry via email and fliers. MBM met mothers and infants at a public park or at their home for sample collection. Due to logistical constraints related to the COVID‐19 pandemic, we enrolled five infants who were older than 6 months. These infants were aged 7.5, 6.8, and 6.5 (*N* = 3 infants) months.

Regardless of recruitment or sampling location, all skin swab samples were collected by rubbing a sterile, dual‐tipped cloth swab (Fisher BD BBL Media‐free Sterile Swab) on each body site for 1 min. Swabs were dipped into a solution of 0.15 M NaCl and 0.1% Tween 20 immediately prior to sample collection. Skin swab samples were collected from infants' hand (palm), axilla, and outer cheek. To minimize contamination from the sampling environment, swabs were applied to the skin upon removal from the sterile plastic containers and returned to the plastic containers immediately after the 1 min of sample collection, at which point the plastic container was stored in a cooler with ice. To confirm that there was no contamination from bacteria in the surrounding air, control samples were taken from each sample collection location by swirling a swab in the air for 1 min. MBM followed COVID‐19 safety protocols throughout the study, including collecting samples outdoors and wearing PPE (face masks and gloves) during interactions with participants. All samples were immediately stored on ice for no more than 5 h before transport to the Amato Laboratory at Northwestern University, where they were stored in a −80° freezer until DNA extraction.

### 2.1 Questionnaires about infants' physical and social environments

At the time of sample collection, mothers completed a detailed questionnaire that addressed their infants' household environments and social interactions with alloparents. MBM designed and administered the questionnaire using the interactive Network Canvas software (Complex Data Collective, 2016), which allowed mothers to generate name‐based social networks for their infants. More specifically, after answering questions about infants' household environments, mothers generated a list of individuals who had “substantial social contact” with their infant since birth (including themselves). Following this section, the questionnaire asked about the types of interactions that each named social contact has with infants “on a typical day.” The questionnaire asked about a set of specific caregiving behaviors: feeding; holding and carrying; playing; skin‐to‐skin contact (defined as both the infant and caregiver partially or fully nude), bathing, and co‐sleeping (defined as napping or sleeping overnight in direct contact with the infant). More details on the questionnaire can be found in Appendices A and B. Throughout this study, the term alloparent is used to refer to any caregivers who are not parents (genetic or social) of the focal infant (Rosenbaum & Gettler, 2018) and were reported to perform at least one caregiving behavior on a typical day.

### 2.2 Microbiome analysis

DNA was extracted from the skin, fecal, and air control samples using the Qiagen DNeasy PowerSoil Pro kit in the Amato Lab at Northwestern University. Extraction modifications for skin samples included warming the CD1 solution and modifying parameters during the vortex and centrifuge steps. The full extraction protocol can be found in Appendix C. The V4‐V5 region of the 16S *rRNA* gene was amplified using a modified version of the Earth Microbiome Project protocol (Thompson et al., 2017) and the 515 Fa/926R primer set (Mallott & Amato, 2018; Walters et al., 2016). Amplicons were barcoded and pooled in equal concentrations for sequencing on the Illumina MiSeq V2 platform at the Genomics and Microbiome Core Facility at the Rush University Medical Center.

Paired‐end sequences were joined and processed using QIIME2 v2020.6 (Bolyen et al., 2019) alongside 88 control samples (a combination of air controls and negative controls from DNA extraction and PCR). Quality filtering and the removal of chloroplast and mitochondria sequences resulted in a total of 11 943 811 reads with an average of 322 180 reads per sample. The dada2 plug‐in was used to cluster amplicon sequence variants (ASVs), and taxonomy was assigned by comparing ASVs to the GreenGenes13_8 reference database. The bacterial composition of the control samples and laboratory negatives were compared to the infant samples. There was no indication of contamination in the infant samples by bacteria from the air, as the bacteria detected in the air samples were of low abundance and/or were not present in the infant samples. Similarly, there was no evidence of contamination in the laboratory negatives. The air control and laboratory negative samples were removed from the final dataset that was used in subsequent statistical analyses.

### 2.3 Statistical analyses

The bacterial diversity (Shannon index) of unrarefied sequences from skin (*N* = 144) and fecal samples (*N* = 27 from infants aged 4–27 weeks) was estimated using log‐ratio modeling in the *DivNet* package (Willis & Martin, 2018) in R (R Core Team, 2021). This package was designed for properties of microbial co‐occurrence network data, including under‐sampling, missing taxa, and overdispersion, and outperforms other methods of diversity estimation that use rarefied data (Willis, 2019). Borrowing from community ecology, diversity represents the taxonomic richness of a sampled bacterial community. Previous work on the infant gut microbiome indicates that diversity fluctuates rapidly in the first weeks to months of life as infants contact myriad bacteria in the physical and social environment (Ferretti et al., 2018). Infant bacterial diversity across the four body sites was visualized in R using the *dabestr* package (Ho et al., 2018).

After estimating bacterial diversity, the *betta* function within the *breakaway* package (Willis et al., 2014) was used to evaluate hypotheses related to diversity estimates. An advantage of constructing regression models with this package is that it reports SEs, allowing for comparisons of effect sizes and confidence intervals, rather than a reliance on *p* values (Berner & Amrhein, 2022; Smith, 2018; Valeggia & Fernández‐Duque, 2022). We deployed hypothesis estimation, as opposed to traditional hypothesis testing, to evaluate our hypotheses based on 95% confidence intervals, effect sizes, and *R* ^2^ values. This is ideal for exploratory studies such as ours, as it allows for a comparison of the ranges of values most compatible with the data (Gelman & Greenland, 2019), avoids binary conclusions about effects based on traditional statistical significance (i.e., *p* values) (Berner & Amrhein, 2022), and promotes biological interpretations of the data (Smith, 2018; Valeggia & Fernández‐Duque, 2022). We first used confidence intervals to assess the possibility that the true association between a given predictor and infant bacterial diversity is zero (i.e., the confidence interval included, or was centered around, zero). For confidence intervals that did not include zero, we then used effect sizes and *R* ^2^ values to compare the relative strength of different predictors and the proportion of variance explained by various models.

In *breakaway*, each regression model used bacterial diversity as the outcome variable and different factors of the social environment as the main predictor. We used correlations and findings from previously published studies to select model covariates. We first explored correlations between variables using the *GGally* package in R (Schloerke et al., 2022), with a particular emphasis on variables that have been associated with the infant microbiome in published studies. Heatmaps of correlations are included in Figure S5. Using Cohen's *d* as a reference for standardized effect sizes, we defined moderate correlation between variables as a Pearson correlation coefficient between .35 and .70, while strong correlation was defined as a coefficient larger than .70. The outcome variable (estimated bacterial diversity) was not moderately or strongly correlated to any other variables. There was moderate correlation between the number of alloparents and birth mode, the presence of male alloparents and breastfeeding status, the presence of female alloparents and having pets in the household, and infant age and birth mode. Additionally, birth mode, infant age, and alloparental feeding were moderately correlated to each other. Alloparental feeding was moderately correlated to infants receiving complementary foods, which was in turn strongly correlated to infant age. Birth mode was moderately correlated to alloparental holding. Maternal care was uniformly robust across mother‐infant dyads, with all mothers reporting to perform each of the caregiving behaviors on a daily basis. Fine‐scale data on maternal care, such as the duration of skin‐to‐skin contact or feeding, was not available to assess potential correlations between maternal care and allocare.

All final models included the following covariates: recent bath (in the 24 h prior to sample collection; binary); Cesarean section delivery (*N* = 6 infants; binary); infant age (in days; continuous); having pets in the household (binary); and current breastfeeding status (binary). Infant sex was not included as a covariate, as it increased the SEs of the models and did not have a clear relationship to bacterial diversity (Figure S3). Household antibacterial product use and infant antibiotic use (during or shortly after delivery) were weakly correlated to infant bacterial diversity and the main predictor variables (Figure S5). One infant was receiving a topical antibiotic during participation in the study, though the medication was not used on the skin sites that were swabbed. Including antibiotic use as a covariate produced very similar results to models that excluded this variable. To avoid overfitting the models, these variables were not included as covariates.

Current infant feeding mode (as reported at the time of sample collection) was categorized by breastfeeding status (binary) in order to capture infants' contact with maternal breast skin during feeding. The “no breastfeeding” group included infants (*N* = 10) whose mothers reported to use a combination of formula, pumped breast milk, and/or formula, but no direct breastfeeding. This categorization allowed for a distinction between infants who are likely exposed to maternal skin bacteria during breastfeeding (even if they also receive pumped milk or complementary foods) versus those who lack the direct exposure to bacteria from maternal breast skin. In addition to evidence that complementary feeding (including formula) is associated with the diversity of the infant gut microbiome (Ho et al., 2018), previous research has detected differences in the composition of pumped breast milk compared to milk that is hand‐expressed for direct breastfeeding (Moossavi et al., 2019). This suggests that pumped breast milk may lack certain bacteria from maternal skin and/or infants' bodies. Since bottle feeding precludes the direct skin‐to‐skin contact between mothers and infants that occurs during nursing, and does not allow for infant suckling to contribute to the bacterial composition of breast milk (Fehr et al., 2020; McGuire & McGuire, 2017; Moossavi et al., 2019), bottle feeding may reduce opportunities for the bidirectional sharing of bacteria between maternal and infant bodies. Due to the known influence of breast milk on the infant gut microbiome, the models of infant fecal bacterial diversity included a covariate that categorized infants based on their exposure to breast milk. The “any milk” group (*N* = 22) included infants who were exposed to breast milk through direct breastfeeding and/or pumped milk. Some of these infants also received formula and/or complementary foods. Infants in the “no milk” group (*N* = 3) were not receiving any breast milk at the time of sample collection.

Regression models addressed three main relationships between infants' social environments and the bacterial diversity of hand, cheek, axilla, and fecal samples. Results were visualized using the *ggforestplot* package (Scheinin et al., 2023). First, models compared the association between the number of alloparents per infant (continuous) and the bacterial diversity of each infant body site (H1). Second, regression models analyzed associations between different alloparent‐infant relationships (binary) and the bacterial diversity of the four infant body sites (H2). The main predictors used in these models were the presence of female adult alloparents (grandmothers, aunts, and non‐relatives); male adult alloparents (grandfathers); and sibling alloparents. These categories were chosen based on the alloparental age and sex classes reported on the maternal questionnaire. Models included only those infants who had one or more alloparent; if infants with zero alloparents were included, it would be unclear if an infant who was in the “no female alloparent” category was also in the “no alloparent” category. Sibling alloparents most often included infants' biological siblings, but in the case of two families, this variable included foster siblings and one cousin. The most frequent behaviors performed by sibling alloparents were playing and holding, which represent opportunities for potential bacterial sharing to infants via direct skin‐to‐skin contact, making them relevant to the overall aims of the study.

Regression models also compared associations between different allocare behaviors and the bacterial diversity of the four infant body sites (H3 and H4). Each allocare behavior was treated as a binary categorical variable (“yes” behavior is performed vs. “no” behavior is not performed). These models did not include bathing or skin‐to‐skin contact (due to insufficient variation in these allocare behaviors in the dataset), and the models of feeding and playing did not include infant fecal samples due to sample size limitations. Questionnaire data on paternal care were included as covariates in the models of allocare; for example, models of alloparental feeding included paternal feeding as a covariate.

Using the *phyloseq* package (McMurdie & Holmes, 2013), relative abundance data were generated for bacterial families that were found in at least 5% of samples with a read count of at least 10 000 across samples. Twenty bacterial families were identified using this threshold and were considered “core” members of the infant microbiome. Relative abundance data were plotted using the *microbiomeutilities* packages (Shetty & Lahti, 2023). This method was used to visualize difference in the core microbiome (H5) of infants based on (1) having adult or (2) sibling alloparents, as well as (3) breastfeeding at the time of sample collection.

## 3 RESULTS

**FIGURE 1:**
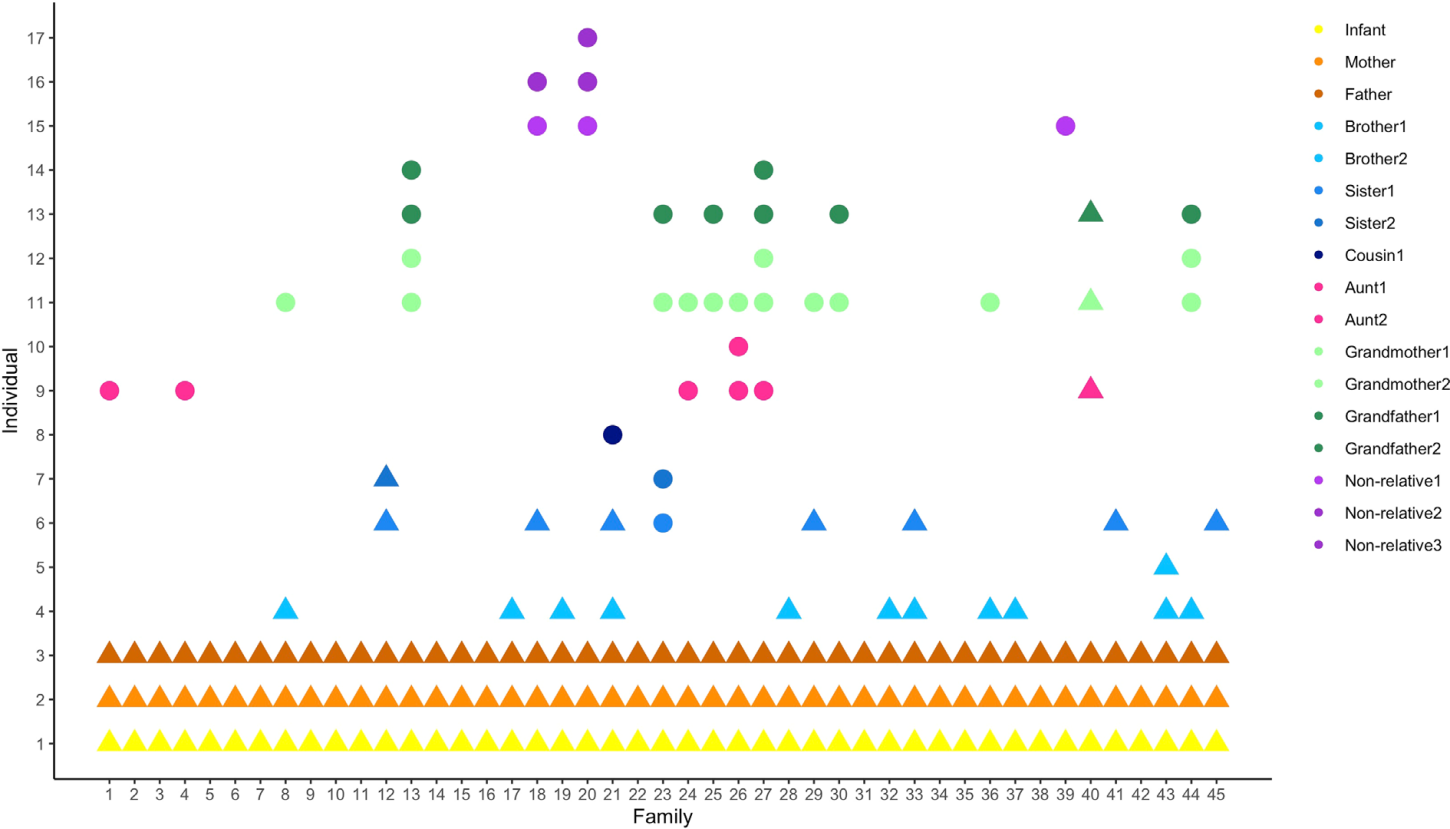
Caregiving structures across study families. Infants are displayed in yellow and parents in orange. All other nodes indicate various alloparents (*N* = 45 families due to one set of twins and two infants with incomplete data). Circles represent individuals who live in a different household from the focal infant, while triangles represent members of the same household.

**FIGURE 2:**
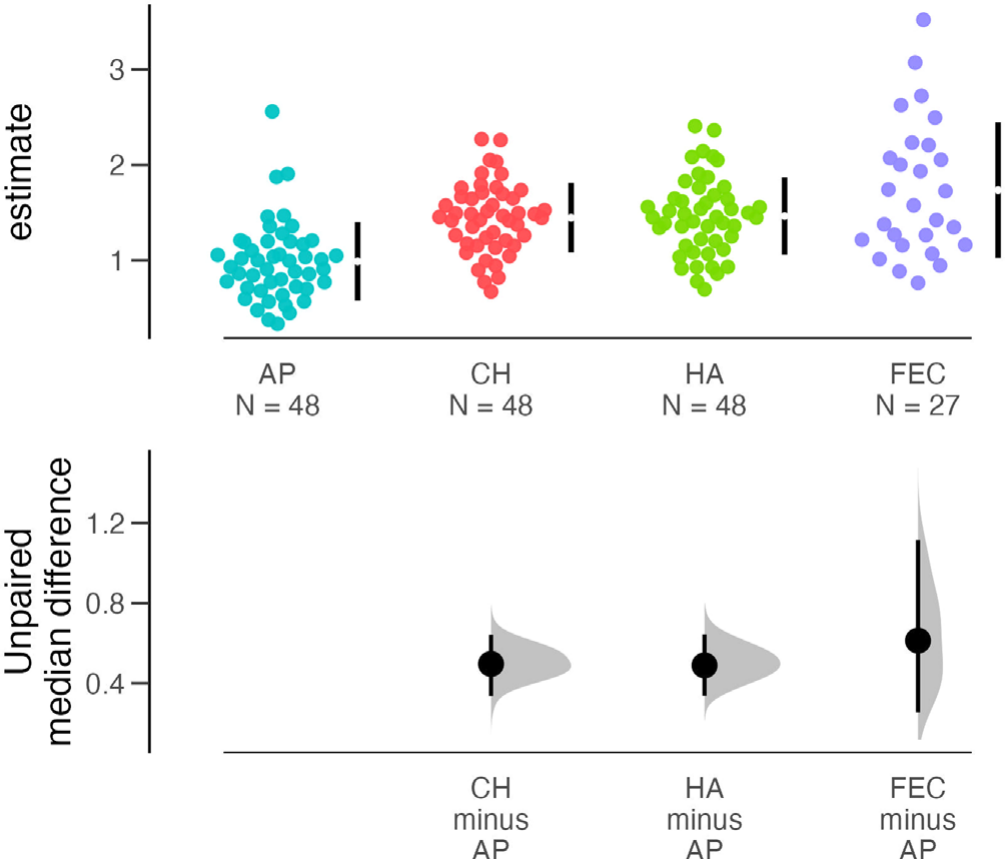
Infant bacterial diversity varies by body site. Vertical bars indicate 95% confidence intervals of the median diversity estimate for each body site. The bottom panel displays the difference in average diversity between each body site compared to the axilla samples. AP, axilla; CH, cheek; Estimate, estimated Shannon diversity using DivNet; FEC, fecal; HA, hand.

This study included 171 hand, axilla, cheek, and fecal samples from 48 infants (including one set of twins) living in Chicago and the surrounding suburbs (Table 1; Figures S1 and S2). The postnatal age of infants ranged from 2 to 30 weeks. All infants lived with their mother and father, who provided regular care. Twenty‐nine (60%) infants had at least one alloparent (Figure 1) and all but one infant lived in a different household than their alloparents (excluding sibling alloparents). Household sizes ranged from two to five people (excluding the infant). Eleven (24%) infants were exclusively breastfed, ten (22%) infants were not breastfeeding, and twenty‐four (53%) infants were breastfeeding and receiving a combination of pumped breast milk, formula, and/or solids (three infants had missing data on current feeding practices). One infant was reported to attend daycare, though due to a COVID‐19 infection at the daycare setting, this infant had not been present in daycare in the days leading up to participation in the study. The diversity of infant bacterial communities differed across the body sites, with axilla samples displaying lower diversity compared to the other body sites (Figure 2). Bacterial diversity did not differ by infant sex (Figure S3). The relative abundances of core bacteria displayed variation by infant body site and age (Figure S4). Fecal samples exhibited a larger range in bacterial diversity compared to the other body sites, yet did not harbor all of the bacterial families that were present in the skin samples.

**TABLE 1:** Demographic data of study participants.

Categorical variables	*N* (%)
Female sex^a^	22 (46%)
C‐section birth	6 (13%)
Pets in the home	32 (67%)
Female alloparents (*N* = 46)	13 (28%)
Male alloparents (*N* = 46)	6 (13%)
Sibling alloparents	20 (42%)
No breastfeeding (*N* = 45)	10 (22%)
Mother works from home^a^	8 (17%)
Recent infant bath (*N* = 47)	16 (34%)

Continuous variables	Mean ± SD (range)
Age (days)	89.11 ± 60.62 (13–212)
Number of alloparents (*N* = 46)	1.37 ± 1.40 (0–5)
Hand bacterial diversity	1.46 ± 0.40 (0.70–2.41)
Cheek bacterial diversity	1.45 ± 0.36 (0.67–2.27)
Axilla bacterial diversity	0.99 ± 0.41 (0.34–2.56)
Fecal bacterial diversity (*N* = 27)	1.74 ± 0.71 (0.76–3.52)

*Note*: Number and percent are shown for categorical variables, and the mean, SD, and range are shown for continuous variables. *N* = 48 infants unless otherwise noted (due to missing data).

^a^ Variables not included in regression models.

### 3.1 Infant bacterial diversity showed non‐zero associations to infant–alloparent relationships, but not the overall number of alloparents

**FIGURE 3:**
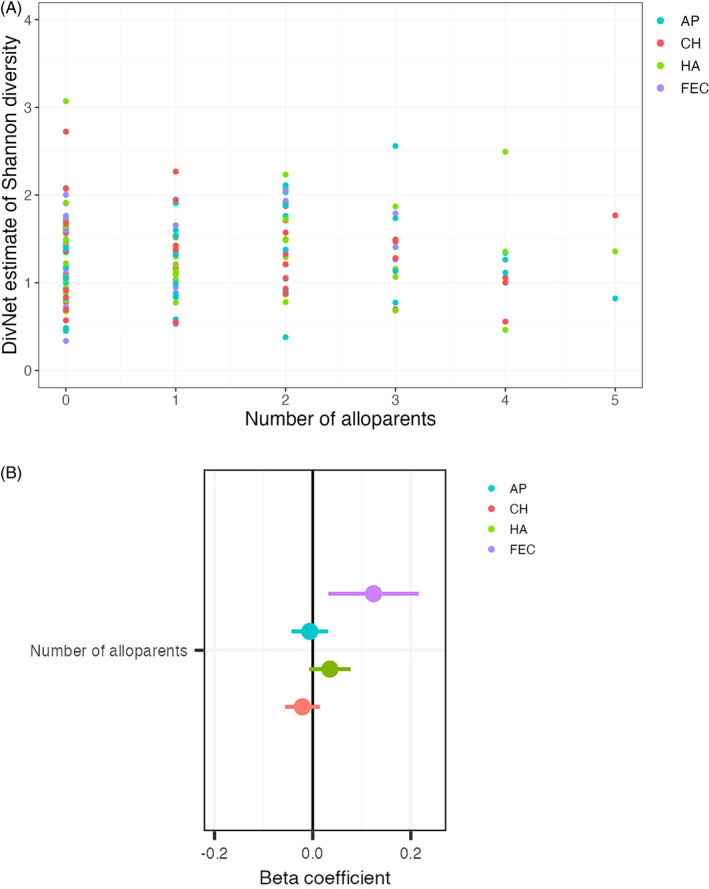
(A) No clear association between the number of alloparents and infant bacterial diversity. (B) The confidence intervals associated with infant skin bacterial diversity were centered around zero. AP, axilla; CH, cheek; FEC, fecal; HA, hand.

The data were not compatible with the hypothesized association between the total number of alloparents and infant bacterial diversity (H1; Figure 3A,B, Table S1). In the models of the three infant skin sites, the confidence intervals centered around zero and included relatively small SEs (Figure 3B). This indicates that the true association between the number of alloparents and infant skin bacterial diversity is likely zero, or a null relationship. While the confidence interval for the model of infant fecal bacterial diversity included positive values, it was wider than those associated with skin bacterial diversity (indicating less model precision) and nearly intersected with zero (0.032–0.216). On average, the effect sizes and *R* ^2^ values for the models that included the number of alloparents as the main predictor were smaller than those that modeled alloparent‐infant relationships and allocare behaviors (Tables S1 and S2).

**FIGURE 4:**
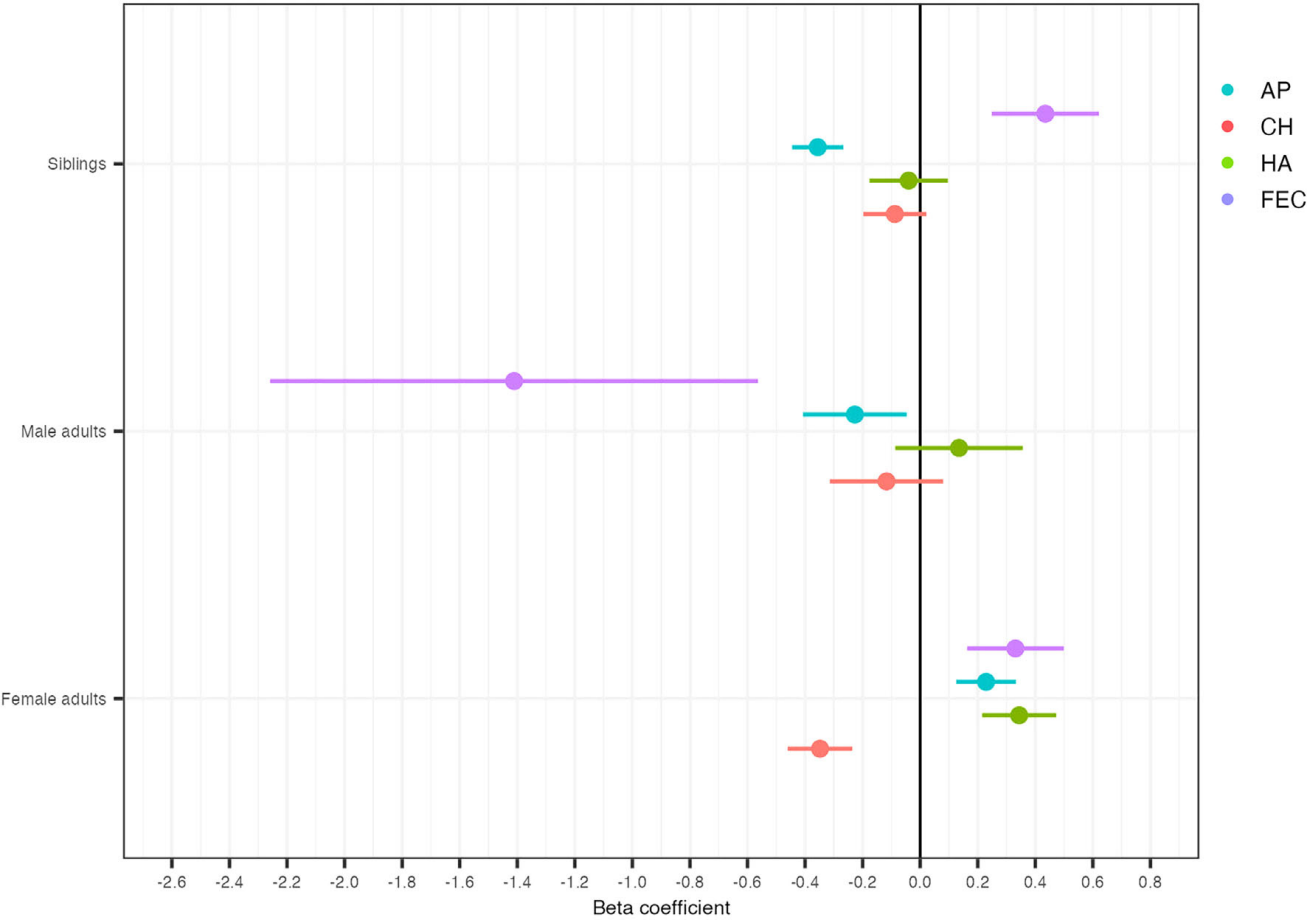
Results of regression models show variable associations between infant–alloparent relationships and infant bacterial diversity (female adults = grandmothers, aunts, and one daycare employee; male adults = grandfathers). Beta coefficients (effect sizes) and 95% confidence intervals are shown for each infant–alloparent relationship. AP, axilla; CH, cheek; FEC, fecal; HA, hand.

In contrast, there were clear associations between infant–alloparent relationships and infant bacterial diversity that were not centered around zero, indicating more compatibility with the second hypothesis (Figure 4, Table S1). Having female adult alloparents was positively associated with infant hand, axilla, and fecal bacterial diversity, and negatively associated with the bacterial diversity of the infant cheek. The *R* ^2^ values were largest for the models of infant axilla (.285) and fecal diversity (.421), and the cheek bacterial diversity model displayed the largest effect size (−.348). In comparison, the ranges of the estimated associations between having male adult alloparents and infant bacterial diversity were larger and often included zero (Figure 4, Table S1). This was particularly true for the model of fecal bacterial diversity, which had low precision, as evidenced by the wide confidence interval. While the sizes of the confidence intervals were equivalent for the three skin sites, only the confidence interval associated with infant axilla bacterial diversity did not include zero. This model also had the largest effect size (−0.227) and *R* ^2^ value (.305) compared to the other skin sites.

The models that estimated the association between having sibling alloparents and the bacterial diversity of the infant cheek and hand also displayed confidence intervals that included zero, while the confidence interval associated axilla bacterial diversity contained only negative values (Figure 4, Table S1). In contrast, the confidence interval for infant fecal bacterial diversity contained only positive values. Accordingly, the *R* ^2^ values and effect sizes were the largest for the models of infant axilla (effect size = −0.356; *R* ^2^ = .402) and fecal (effect size = 0.435; *R* ^2^ = .671) bacterial diversity compared to the other two body sites.

### 3.2 Allocare behaviors displayed variable associations with infant bacterial diversity

**FIGURE 5:**
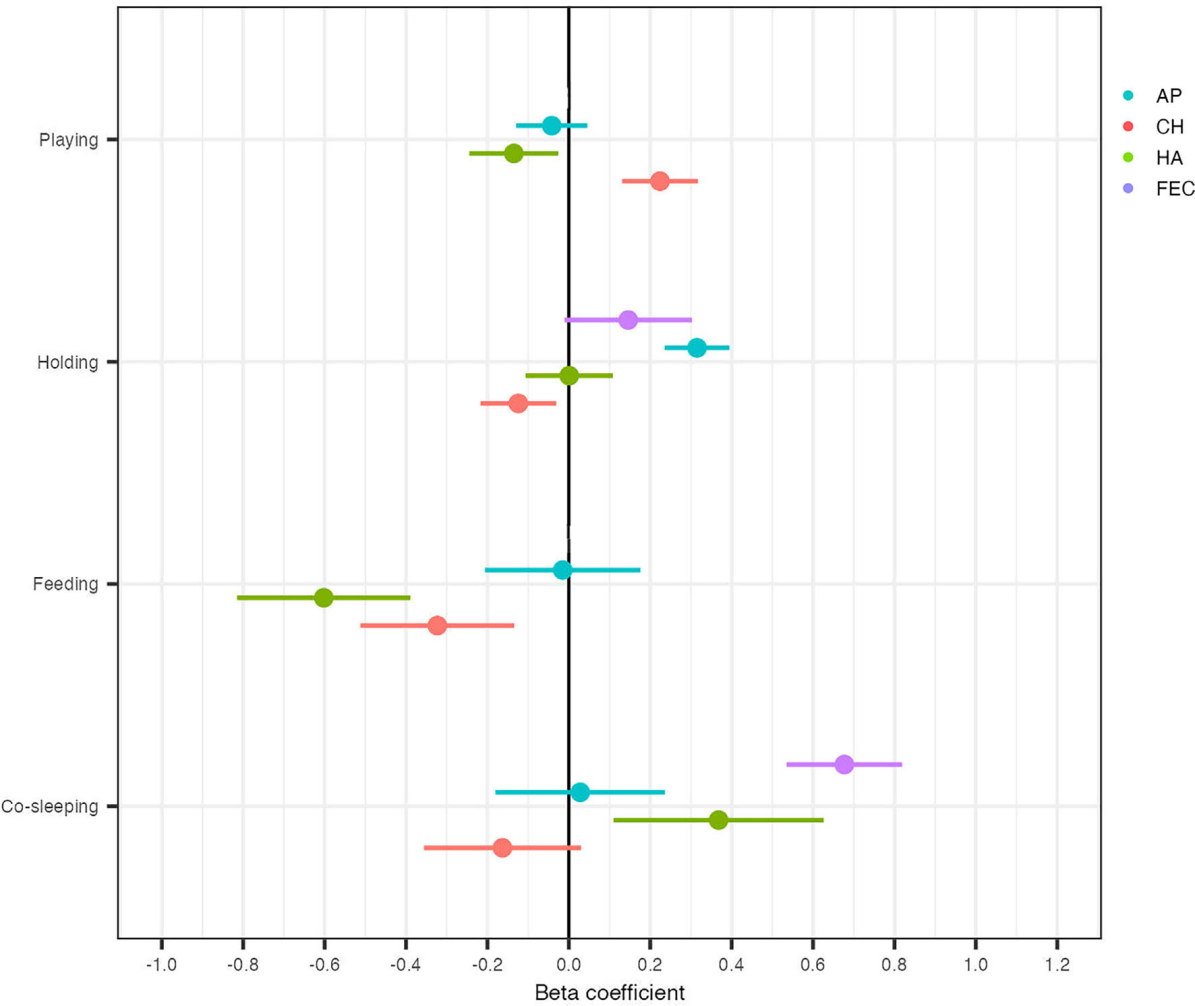
Results of regression models show variable associations between allocare behaviors and infant bacterial diversity. Beta coefficients (effect sizes) and 95% confidence intervals are shown for each infant–alloparent relationship. AP, axilla; CH, cheek; FEC, fecal; HA, hand.

Results of regression models indicated that allocare behaviors exhibited different relationships with bacterial diversity across the infant body sites (H3 and H4). Alloparental feeding was strongly negatively associated with the bacterial diversity of the infant hand and cheek, while the estimated association to axilla bacterial diversity was centered around zero (Figure 5, Table S2). Alloparental co‐sleeping had a strong, positive relationship to infant hand and fecal bacterial diversity, and the estimated association was largest for fecal diversity (effect size = 0.677; *R* ^2^ = .904). The confidence interval associated with infant cheek bacterial diversity included mostly negative values (as well as zero), and the confidence interval associated with infant axilla bacterial diversity was centered around zero.

The confidence interval associated with alloparental holding and the bacterial diversity of infant hand and fecal samples also included zero (Figure 5, Table S2). There was a negative association between this caregiving behavior and cheek bacterial diversity, and a stronger positive association with axilla bacterial diversity (effect size = 0.315; *R* ^2^ = .307). Alloparental play had a non‐zero association with both infant hand and cheek bacterial diversity, though the direction of these relationships differed (negative and positive, respectively). The relationship was strongest to infant cheek bacterial diversity (effect size = 0.224; *R* ^2^ = .187).

### 3.3 The relative abundances of core bacterial families varied by infant–alloparent relationship and breastfeeding status

**FIGURE 6:**
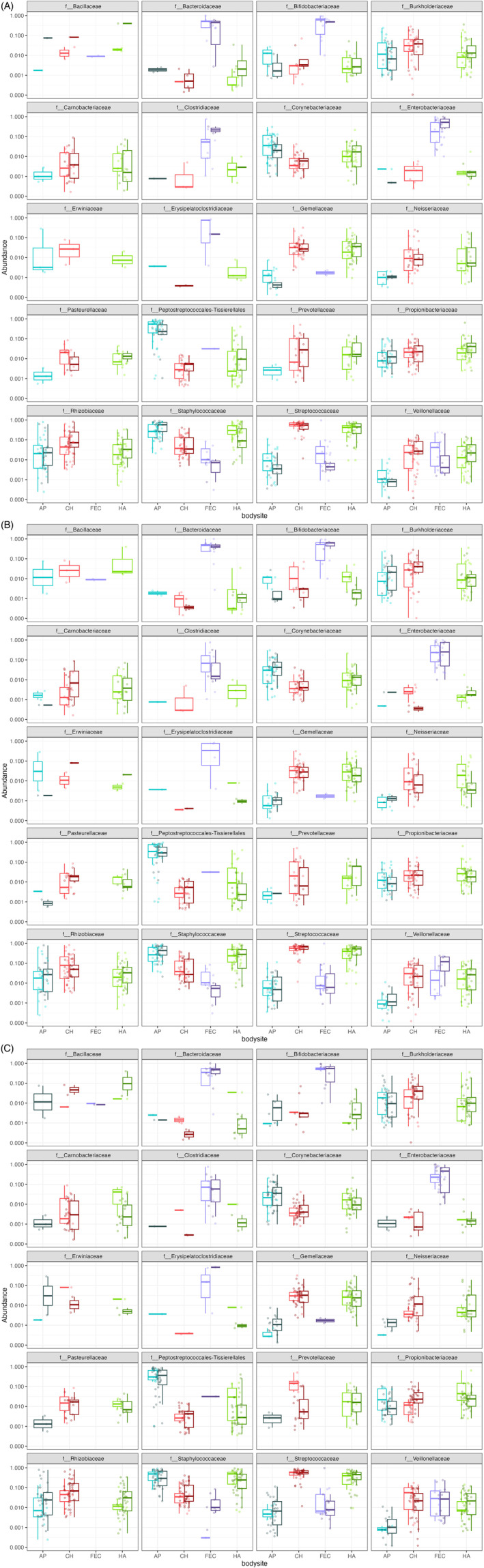
The relative abundances of core bacterial families varied based on having (A) adult and (B) sibling alloparents, as well as (C) breastfeeding status. In each plot, the x‐axis displays the four infant body sites. Infants with adult and sibling alloparents, and those who were breastfed, are represented by the darker shade of each color. AP, axilla; CH, cheek; FEC, fecal; HA, hand.

The data indicated variation in the relative abundances of bacterial families across infant body sites and in relation to allocare (H5; Table S3, Figure 6). Of the 20 “core” bacterial families, not all were detected in samples from each of the infant body sites (Figure S4B). Nine bacterial families were detected in infant skin samples but not fecal samples, while other bacterial families were found only in a small number of samples for a given body site. The relative abundances of certain bacterial families (e.g., *Enterobacteriaceae* in fecal samples and *Veillonellaceae* in hand samples) appeared to be elevated in the samples of infants who had adult alloparents, compared to their counterparts without adult alloparents (Figure 6A). Other taxa (e.g., *Corynebacteriaceae* in axilla samples and *Rhizobiaceae* in hand samples) appeared elevated in the samples of infant who had sibling alloparents compared to those who did not (Figure 6B). In contrast, infants with adult alloparents displayed lower relative abundances of *Peptostreptococcales–Tissierellales* in their axilla samples, just as the hand and cheek samples of infant with sibling alloparents showed reduced relative abundances of *Neisseriaceae* and *Prevotellaceae*, respectively.

There was also variation in the relative abundances of bacterial families based on breastfeeding status (Figure 6C). Compared to infants who were not breastfeeding at the time of sample collection, the hand samples of breastfed infants displayed lower relative abundances of multiple taxa, including *Propionibacteriaceae*, *Carnobactieraceae*, and *Peptostreptococcales–Tissierellales*. In contrast, the relative abundances of these same taxa were higher in the cheek samples of breastfed infants. Certain taxa displayed differential relative abundances in some, but not all, of the body sites. For example, *Veillonellaceae* appeared to vary by breastfeeding status in infant axilla, cheek, and hand samples to a greater degree than in fecal samples. The relative abundance of *Prevotellaceae* was lower in the cheek samples of breastfed infants but did not differ in hand samples.

Trends in bacterial relative abundances were not uniform when comparing across the presence of alloparents versus breastfeeding status. As one example, the relative abundance of *Rhizobiaceae* was elevated in axilla samples in relation to breastfeeding to a much greater degree than in relation to having sibling alloparents. The relative abundance of this taxon in infant axilla samples did not appear to vary based on having adult alloparents. Similarly, the relative abundance of *Propionbacteriaceae* in infant cheek samples did not vary based on having adult or sibling alloparents, yet was much higher in the cheek samples of breastfed infants.

## 4 DISCUSSION

This study explored the relationship between allocare and the diversity of infant skin and fecal bacterial communities. Given the novel nature of this dataset, one goal of this study was to compare associations between bacterial diversity and different components of allocare across multiple infant body sites. Only infant fecal bacterial diversity displayed a non‐zero association to the number of alloparents, providing mixed support for our first hypothesis. In line with our second hypothesis, infant bacterial diversity showed stronger associations to the presence of female adult alloparents compared to sibling or male adult alloparents. Infant bacterial diversity also varied in relation to particular allocare behaviors and body sites (H3 and H4). Many of these associations were in opposing directions, suggesting that the relationship between allocare behaviors and the diversity of infant bacterial communities varies by infant body site. The relative abundances of bacterial families varied by infant–alloparent relationship and breastfeeding status (H5). Since infant bacterial diversity (particularly on the skin) was more strongly associated with infant–alloparent relationships and allocare behaviors, rather than overall number of alloparents, our findings suggest that continuous measures of social environments may be inadequate for capturing the nuanced behaviors that drive infant–alloparent interactions and by extension, opportunities for bacterial sharing (Lane et al., 2019). By evaluating alloparent–infant relationships, as well as specific allocare behaviors, this study provides some of the first evidence that allocare is differentially associated with the diversity of bacterial communities across the infant skin and gut.

### 4.1 Female adult alloparents displayed the strongest associations with infant bacterial diversity

The bacterial diversity of all four infant body sites had non‐zero associations to having female adult alloparents. The strength of the relationships was similar across the four body sites, though the association was negative in relation to cheek bacterial diversity. It is possible that infant cheek bacterial diversity is influenced by direct contact with female adult alloparents through behaviors such as kissing. Female adult alloparents may also be a proxy for unmeasured factors that have the potential to shape infant cheek bacterial communities. For example, female adult alloparents who take infants outdoors may indirectly influence the bacterial communities of infant skin sites that are more exposed to the surrounding environment (i.e., cheek), compared to those that are covered by clothing or blankets (i.e., axilla and hand).

The relatively narrow confidence intervals associated with these models may reflect the prevalence of female adult alloparents in this study (the precision of estimated associations is sensitive to sample size), as well as the types of caregiving behaviors performed by female alloparents. Female adults were the only alloparent reported to feed infants. In this study, this behavior was synonymous with non‐exclusive breastfeeding, which is known to affect the diversity of the infant gut microbiome (Ho et al., 2018). Though we could not directly test for this association with infant fecal diversity due to missing data (i.e., no fecal samples were collected from infants whose female adult alloparents were reported to feed), alloparental feeding showed a strong negative relationship to infant cheek and hand bacterial diversity. Infant feeding is often followed by washing infants' cheeks and hands—a practice that impacts skin bacterial communities (Korting et al., 1987; Yu et al., 2018) and may contribute to the observed lower bacterial diversity in infants who were fed by female adult alloparents. In contrast, multiple confidence intervals attributed to having male adult alloparents contained zero. This may have been due to sample size limitations, as few infants in the study had male adult alloparents, though it may also reflect genuine sex‐specific differences in allocare that impact the infant microbiome (Manus et al., 2023; Rosenbaum et al., 2021).

### 4.2 Having sibling alloparents was associated with the diversity and composition of bacterial communities across the infant body

Infants with sibling alloparents displayed elevated fecal bacterial diversity compared to their counterparts without sibling alloparents. This may reflect infants' bacterial exposures that stem from touching their siblings and putting shared objects, including toys and food items, into the mouth (Manus, 2021; Shaffer & Lozupone, 2018). It is also possible that sibling alloparents, the majority of which were between two and five years old, helped expose infants to bacteria that originated in daycare or preschool settings. The elevated fecal diversity of infants with siblings may be correlated to the observed lower relative abundances of *Clostridiaceae*; this bacterial family contains several pathogens that could be excluded from diverse gut communities through competitive ecological interactions between resident bacteria and invading pathogens (Midlej & Benchimol, 2010; Theriot et al., 2014).

Our findings expand on the literature linking siblings to the diversity of the infant gut microbiome (Azad et al., 2013; Hasegawa et al., 2017) by demonstrating associations to infant skin bacterial communities. As one example, we detected lower relative abundances of *Neisseriaceae* in the hand samples of infants with sibling alloparents, while this bacterial family did not appear to vary in relation to having adult alloparents. Bacteria in the *Neisseriaceae* family are known contributors to the oral microbiome (Ye et al., 2022) and different species can either prevent or lead to upper respiratory tract infections during infancy (Theodosiou et al., 2022). Additional studies are needed to explore potential transmission routes of this taxon between the oral and skin bacterial communities of infants and their sibling alloparents.

### 4.3 Infant axilla bacterial diversity displayed unique associations with allocare

The axilla was the only body site to display a positive, non‐zero association between bacterial diversity and alloparental holding, a behavior commonly performed by female adult alloparents (as indicated on the maternal questionnaire). Accordingly, we detected a similar positive relationship between infant axilla bacterial diversity and having female adult alloparents. In contrast, the confidence intervals that associated axilla bacterial diversity to the other allocare behaviors were centered around zero. This could be due to the fact that compared to other skin sites, axillae are not as frequently directly contacted by alloparents during bouts of feeding, playing, or co‐sleeping. Instead, axillae may be exposed to a suite of environmentally sourced bacteria on alloparents' hands (Ross et al., 2017; Shaffer & Lozupone, 2018) while being picked up and held. Behavioral observations of infants and alloparents (Meehan et al., 2018) are needed to confirm patterns of contact with infant axillae and other skin sites. Unlike the associations with having female adult alloparents, infant axilla bacterial diversity was negatively associated with having sibling and male adult alloparents. This suggests that alloparents of different ages and sexes may have opposing influences on the bacterial diversity of the infant axilla, potentially due to differences in the behaviors that put them into contact with this body site. In support of this idea, the relative abundance of certain taxa, including *Burkholderiaceae*, in axilla samples showed a positive relationship to having sibling alloparents, yet a negative relationship to having adult alloparents.

The bacterial diversity of the infant axilla was lower than that of the cheek and hand, a finding in line with studies of the adult skin microbiome (Manus et al., 2017; Perez et al., 2016). The physiology of infant axillary skin likely influences the types of bacteria that can successfully colonize the body site, potentially regardless of the frequency of potential bacterial transmission events from alloparents (Manus, 2021). Studies in adults suggest that the lower bacterial diversity of the axilla (Manus et al., 2017; Perez et al., 2016) is due to the use of hygiene products (Urban et al., 2016; Yu et al., 2018) in tandem with local ecological properties, including moisture and sebum, that promote the growth of certain bacteria while restricting others (Byrd et al., 2018). While infant and adult skin display different physiological properties (Nikolovski et al., 2008) and are exposed to different hygiene products, local conditions within the infant axilla might “screen” which environmentally sourced bacteria are able to successfully colonize the skin site (Manus, 2021)—a process known as *ecological selection* (Rawls et al., 2006).

### 4.4 Alloparental co‐sleeping and feeding showed the strongest associations to infant bacterial diversity

Co‐sleeping showed a strong positive relationship to infant fecal and hand bacterial diversity. In our study, this behavior included bouts of napping in which alloparents were in direct contact with infants. Without observational data, it is unclear if co‐sleeping was correlated with an increase in other practices known to influence the infant gut microbiome, such as feeding (Gettler & McKenna, 2011). It may be that infants fall asleep while being fed by their alloparents, or similarly, infants who sleep in close proximity to alloparents are more likely to be fed immediately upon waking. Both scenarios could help explain the strong association between co‐sleeping and infant fecal bacterial diversity. Napping could also include incidental contact with older siblings or pets, two sources of bacterial exposures for infants (Azad et al., 2013; Hasegawa et al., 2017; Lane et al., 2019; Laursen et al., 2017).

Alloparental feeding was associated with decreased bacterial diversity of the infant hand and cheek (with the strongest relationship to the bacterial diversity of the hand). Intriguingly, hand samples of infants who were not breastfed at the time of data collection appeared to harbor elevated relative abundances of the bacterial family *Carnobacteriaceae*. Since *Carnobacteriaceae* are commonly found in packaged and preserved foods (Hansen et al., 2021), it may be that infants who were not breastfed had a greater exposure to *Carnobacteriaceae* in formula, solid foods, or the bodies of alloparents who participated in feeding. Infant hands may also be exposed to this bacterial family (and others) directly from bottles, particularly if bottles harbor bacteria from adult food items or cutlery that are used, cleaned, and stored in a shared kitchen. Samples of the household bacterial environment, including from kitchen surfaces, would help clarify the origin of bacteria like *Carnobacteriaceae* within the household. Since the breastfeeding category used in this study was non‐exclusive, breastfed infants may have been similarly exposed to bacteria from the household bacterial environment through complementary feeding. This could explain why *Carnobacteriaceae* was also detected on the hands of breastfed infants (though at a lower relative abundance compared to non‐breastfed infants). Of note, this taxon was not detected in infant fecal samples, which suggests that exposure to this bacterium may be limited to contact with the skin in our study population. We also detected elevated relative abundances of *Enterobacteriaceae* in the fecal samples of breastfed infants. This may stem from increased exposure to bacteria in this family that originate on maternal breast skin (Fryklund et al., 1995; Rahimzadeh Torabi et al., 2021).

### 4.5 Strengths, limitations, and future directions

This study contributes to the literature that connects social environments and bacterial diversity across a range of host species, including humans (Brito et al., 2019; Dill‐McFarland et al., 2019), non‐human primates (Moeller et al., 2016; Perofsky et al., 2017), bats (Kolodny et al., 2019), and insects (Kwong and Moran, 2016). We expand on previous studies that used the number of household members or caregivers as indicators of infants' social environments (Azad et al., 2013; Lane et al., 2019; Manus et al., 2023) by modeling various infant–alloparent relationships and specific allocare behaviors. Another strength of the current study is the use of hypothesis estimation (Berner & Amrhein, 2022; Gelman & Greenland, 2019) instead of traditional testing to assess the compatibility of the data to our hypotheses. This method is well suited for studies in human biology that aim to interpret the biological significance of associations using relatively small sample sizes (Smith, 2018; Valeggia & Fernández‐Duque, 2022).

By collecting infant skin and fecal samples, we highlighted trends in bacterial diversity and composition across multiple body sites that can be used to guide future studies toward quantifying bacterial transmission events. For example, we detected *Rhizobiaceae* in samples collected from the three infant skin sites, but not in the fecal samples. Since this bacterial family is typically associated with plant and soil microbiomes, yet has also been found in households (Rintala et al., 2008), its presence on infant skin highlights the need to explore bacterial transmission events within infants' natural and built environments. Collecting microbiome samples from different household surfaces will help clarify if similarities between infants and alloparents are due to coexisting within a shared indoor environment (Ross et al., 2017; Song et al., 2013).

The current study also expands on previous research linking infant feeding mode to variation in the breastmilk and infant gut microbiomes (Cioffi et al., 2020; Fehr et al., 2020; Moossavi et al., 2019), and highlights the utility of exploring the skin microbiome in relation to infant feeding and health (Gale et al., 2012). Continued research on feeding practices during in early life is needed to better understand the window of infancy during which the microbiome is most sensitive to acquiring new community members from caregivers' bodies and other environmental sources. Initial exposure to a certain quantity or type of maternal bacteria could affect the ability of bacteria from other sources, including alloparents, to colonize the infant body. This ecological phenomenon, known as *priority effects*, may set the stage for subsequent bacterial community development, including how a given bacterial community responds to future disturbances (Allison & Martiny, 2008; Coyte et al., 2019; Debray et al., 2021). Given the widespread interest in infant feeding across the fields of anthropology, public health, and nutrition (Bridgman et al., 2017; Gale et al., 2012; McKenna et al., 1997; Pannaraj et al., 2017; Thompson & Bentley, 2013; Wilkinson et al., 2021), studying the interplay between allocare, infant feeding practices, and the infant microbiome has implications for improving pediatric care and policy recommendations related to infant health.

A limitation of the current study is the use of *16S rRNA* bacterial gene sequencing, which does not provide the robust taxonomic resolution needed to infer bacterial sharing between infants and alloparents. Future studies will benefit from sequencing approaches that begin to untangle transmission dynamics of specific bacterial species and strains, such as whole genome sequencing. This will help quantify bacterial sharing between alloparents and different infant body sites (Korpela et al., 2018; Meisel et al., 2016), including the potential for bidirectional bacterial transfer. Expanding on ideas related to the retrograde inoculation hypothesis, which posits that the bacterial composition of breastmilk may be shaped by the infant oral microbiome (McGuire & McGuire, 2017; Moossavi et al., 2019), bacteria may also be spread from infant skin to the bodies of their alloparents.

The cross‐sectional design of the current study limited our ability to infer patterns of infant microbiome development. Longitudinal samples are sorely needed to understand the temporal dynamics of bacterial exposures in early life, including daily fluctuations in bacterial diversity. Future studies will ideally couple longitudinal microbiome samples with biomarkers of immune development in order to shed light on the pathways that connect social environments, the microbiome, and infant health. While this study did not collect self‐reported data about race, ethnicity, or cultural background, researchers should explore how these demographics shape infant caregiving dynamics, as well as how racism and discrimination contribute to inequitable bacterial exposures on both the individual and community level (Amato et al., 2021; Ishaq et al., 2019, 2021). Given the timing of this study, it is likely that caregivers' behavioral responses to the COVID‐19 pandemic, including social distancing, masking, and increased hand hygiene, affected infants' social and bacterial exposures in ways that were not captured by our questionnaire. Finally, it is also noteworthy that allocare may be negatively correlated to maternal care in certain contexts. Future studies should consider how additional components of early life biosocial development (other than the microbiome) may be affected by this tradeoff.

Continuing to explore the infant microbiome in an ecological and evolutionary context can strengthen our understanding of the role that bacteria played throughout human evolution (Amato, 2017; Amato et al., 2019; Davenport et al., 2017). While this study was underpowered to test the effects of different allocare behaviors within specific infant–alloparent relationships, replicating our approach across additional populations has the potential to inform broader anthropological theory. This includes the “Grandmother Hypothesis,” which posits that extended human longevity allows post‐menopausal women opportunities to continue to assist their kin (Hawkes et al., 1998). In addition to well‐studied grandmaternal behaviors like food provisioning (Hawkes et al., 1997) and enhancing children's emotional and cognitive development (Awad & Sonuga‐Barke, 1992; Pope et al., 1993), bacterial sharing offers a novel perspective on the pathways through which grandmothers, and other family members, may promote infant survival (Hill & Hurtado, 2009) and maternal wellbeing (Sear et al., 2003). The current study provides some of the first evidence that the infant skin microbiome can be leveraged as an additional tool for studying the effects of cooperative breeding and allocare on infant physiology and health (Lane et al., 2019; Meehan et al, 2018). Continued work across human populations, as well as comparative studies in other cooperative breeding primates, such as marmosets and tamarins (Garber, 1997), can help shed light on the evolutionary dynamics of infant–alloparent interactions and associated bacterial sharing.

## 5 CONCLUSION

The current study joins the burgeoning literature on the environmental influences on the skin microbiome of healthy infants (Capone et al., 2011; Chu et al., 2017; Manus et al., 2020) and provides some of the first empirical evidence that infant fecal and skin bacterial diversity is associated with the social environment. We present novel data that couples microbiome samples with detailed information on infant–alloparent relationships and specific allocare behaviors, which revealed many opposing associations between allocare and the bacterial diversity of different infant body sites. This study lays the foundation for investigating how socioeconomic and cultural forces can affect infants' social and bacterial environments. For example, global variation in parental leave policies may engender disparities in early life caregiving environments, and by extension, infants' bacterial exposures. Within this framework, the influence of allocare on the infant microbiome represents an additional dimension of the social determinants of health. Future research that collects health biomarkers alongside microbiome samples will help characterize the early life “critical window” of infancy (Kuzawa & Quinn, 2009; Stinson, 2019) during which human biology is particularly sensitive to inputs from the surrounding social and bacterial environment.

## AUTHOR CONTRIBUTIONS

*Conceptualization*, *data curation*, *formal analysis*, *funding acquisition*, *investigation*, *methodology*, *project administration*, *software*, *visualization*, *writing* (*original draft preparation*): Melissa B. Manus. *Investigation*, *methodology*, *project administration*, *resources*, *supervision*, *writing* (*reviewing and editing*): Maria Luisa Savo Sardaro. *Investigation*: Omolola Dada. *Investigation*: Maya I. Davis. *Investigation*, *writing* (*reviewing and editing*): Melissa R. Romoff. *Investigation*: Stephanie G. Torello. *Investigation*: Esther Ubadigbo. *Investigation*: Rebecca C. Wu. *Resources*, *software*, *supervision*, *writing* (*reviewing and editing*): Emily S. Miller. *Conceptualization*, *resources*, *supervision*, *writing* (*reviewing and editing*): Katherine R. Amato.

## CONFLICT OF INTEREST STATEMENT

The authors declare no conflict of interest.

## Supporting information


**FIGURE S1.** Details of participant recruitment from four different locations.

**FIGURE S2.** Location of study families' residential zip codes across Chicagoland. Blue circles indicate the number of families sampled from a given area, which are labeled by median household income (red = lower; green = higher). This original image was created by Joanna B.B. Simon using publicly available 2019 United States Census data.

**FIGURE S3.** Infant bacterial diversity did not show a clear relationship to infant sex. Vertical bars indicate 95% confidence intervals of the median diversity estimate for each group of samples. The bottom panel displays the difference in average diversity between female and male infants. Estimate = estimated Shannon diversity using DivNet; M = male; F = female; AP = axilla; CH = cheek; FEC = fecal; HA = hand.

**FIGURE S4.** The relative abundances of core bacterial families by (a) infant age and (b) body site. In each plot, the x‐axis displays the four infant body sites. Infant age categories are represented by shading (lighter shade = 0–3 months; middle shade = 3–6 months; darker shade = 6+ months). AP = axilla; CH = cheek; FEC = fecal; HA = hand.

**FIGURE S5.** Heatmaps illustrating correlations between infant bacterial diversity, environmental variables, and (a) infant‐alloparent relationships or (b) allocare behaviors.

**TABLE S1.** Results of regression models displaying associations between infant bacterial diversity and (a) number of alloparents and having (b) female adult alloparents, (c) male adult alloparents, and (d) sibling alloparents. Effect = beta coefficient; SE = standard error; LCI = lower bound of 95% confidence interval; UCI = upper bound of 95% confidence interval. In tables b‐d, models excluded infants with no reported alloparents. CH = cheek; HA = hand; AP = axilla; FEC = fecal.

**TABLE S2.** Results of regression models displaying associations between infant bacterial diversity and alloparental (a) feeding, (b) co‐sleeping, (c) holding, and (d) playing. Effect = beta coefficient; SE = standard error; LCI = lower bound of 95% confidence interval; UCI = upper bound of 95% confidence interval. CH = cheek; HA = hand; AP = axilla; FEC = fecal.

**TABLE S3.** Relative abundance data for core bacterial families in infant samples.

## Data Availability

The data that support the findings of this study are openly available in SRA at https://www.ncbi.nlm.nih.gov/sra (BioProject Accession PRJNA1004334).

## APPENDIX A

### QUESTIONS FROM THE MATERNAL QUESTIONNAIRE THAT WERE ADMINISTERED WITH THE NETWORK CANVAS SOFTWARE

1. Mother's name, contact information, and zip code
2. Infant's name and DOB
3. Did you take any antibiotics during your pregnancy?
4. Did you or your infant receive any antibiotics during or soon after labor?
5. If you or your infant are currently taking antibiotics, please report which type
6. Was your infant delivered vaginally or via Cesarean section?
7. Are you practicing skin‐to‐skin contact with your infant?
8. Excluding your infant, how many people live in your household?
9. How many rooms are in your home (excluding bathrooms)?
10. Excluding your infant, how many children live in your household?
11. Have you been working from home for any time since March 2020?
12. If you have pets in your home, indicate the number of each type
13. Which feeding methods have you used since your infant was born? (choose all that apply): Breastfeeding; Formula feeding; Pumping and bottle feeding; Solids/complementary foods
14. Which feeding methods are you currently using? (choose all that apply): Breastfeeding; Formula feeding; Pumping and bottle feeding; Solids/complementary foods
15. Where does your infant typically sleep (choose all that apply): Alone in separate room; In my room but not in my bed; In my bed with me; Other
16. How frequently does your infant receive a bath?: Less than once per week; Once or twice per week; 3–6 times per week; Daily
17. Did your infant receive a bath in the last 24 h?
18. List which products your use on your infant's skin:
19. Did you bathe in the last 24 h?
20. List which products your use on your skin:
21. List which cleaning products are regularly used in your home:
22. Besides yourself, has anyone else provided substantial care to your infant since they were born?
23. Who has your infant come into contact with since birth? Consider individuals who have had substantial social contact with your infant (e.g., friends, family members, daycare providers; not individuals with sporadic contact, such as a doctor)
24. For each named individual, please list their biological sex and age:
25. For each named individual, what is their relationship to your infant?: Parent; Grandparent; Aunt or uncle; Cousin; Sibling; Non‐relative
26. For each named individual, does this person live in the same home as your infant?
27. For each named individual, is this per a primary caregiver, secondary caregiver, or occasional contact for your infant?
28. For each named individual, how much time do they spend in physical contact with your infant on a typical day?: No contact; <1 h; 1–5 h; 5–10 h, 10+ h
29. For each named individual, how much time did they spend in physical contact with your infant yesterday?: No contact; <1 h; 1–5 h; 5–10 h, 10+ h
30. For each named individual, do they provide overnight care to your infant on a typical night?
31. For each named individual, do they feed your infant on a typical day?
32. For each named individual, did they feed your infant in the last 24 h?
33. For each named individual, do they bathe your infant on a typical day?
34. For each named individual, did they bathe your infant in the last 24 h?
35. For each named individual, do they hold/carry your infant on a typical day?
36. For each named individual, did they hold/carry your infant in the last 24 h?
37. For each named individual, do they play with your infant on a typical day?
38. For each named individual, did they play with your infant in the last 24 h?
39. For each named individual, do they co‐sleep with your infant on a typical day?
40. For each named individual, did they co‐sleep with your infant in the last 24 h?
41. For each named individual, do they practice skin‐to‐skin contact with your infant on a typical day?
42. For each named individual, did they practice skin‐to‐skin contact with your infant in the last 24 h?

## APPENDIX C

### MODIFIED LABORATORY PROTOCOL FOR BACTERIAL DNA EXTRACTION FROM SKIN SWAB SAMPLES USING THE QIAGEN DNeasy PowerSoil Pro Kit

1. Spin the PowerBead Pro Tube briefly to ensure that the beads have settled at the bottom. Add the skin swab head and trim the stem with sterilized scissors. Add 800 μL of Solution CD1. Vortex briefly to mix and heat at 70°C for 15 min.
2. Secure the PowerBead Pro Tube horizontally on a Vortex adapter and vortex at maximum speed for 15 min.
3. Centrifuge the PowerBead Pro Tube at 13 000 × g for 1 min.
4. Transfer the supernatant to a clean 2 mL Microcentrifuge Tube.
5. Add 200 μL of Solution CD2 and vortex for 10 s.
6. Put tubes into 4°C fridge for 10 min.
7. Centrifuge at 13 000 × g for 1 min. Avoiding the pellet, transfer up to 700 μL of supernatant to a clean 2 mL Microcentrifuge Tube.
8. Add 600 μL of Solution CD3 and vortex for 5 s.
9. Load 650 μL of the lysate onto an MB Spin Column and centrifuge at 13 000 × g for 30 s.
10. Discard the flow‐through and repeat step 9 to ensure that all of the lysate has passed through the MB Spin Column.
11. Place the MB Spin Column into a clean 2 mL Collection Tube.
12. Add 500 μL of Solution EA to the MB Spin Column. Centrifuge at 13 000 × g for 1 min.
13. Discard the flow‐through and place the MB Spin Column back into the same 2 mL Collection Tube.
14. Add 500 μL of Solution C5 to the MB Spin Column. Centrifuge at 13 000 × g for 1 min.
15. Discard the flow‐through and place the MB Spin Column back into a new 2 mL Collection Tube.
16. Centrifuge at 13 000 × g for 2 min. Place the MB Spin Column into a new 1.6 mL Elution Tube.
17. Add 80 μL of Solution C6 to the center of the white filter membrane.
18. Centrifuge at 13 000 × g for 2 min. Discard the MB Spin Column. Store the DNA in the fridge or freezer.
