# Supplementary figures and images for: Interactions with alloparents are associated with the diversity of infant skin and fecal bacterial communities in Chicago, United States
Source: Am J Hum Biol. 2023 Aug 26;37(1):e23972. doi: 10.1002/ajhb.23972 (PMC11667966; doi:10.1002/ajhb.23972)

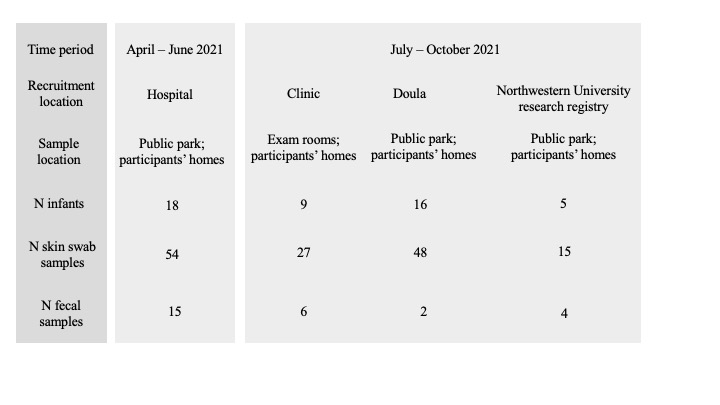

Supplement: Supplementary file 1 — FIGURE S1. Details of participant recruitment from four different locations. [file AJHB-37-e23972-s001.jpeg]

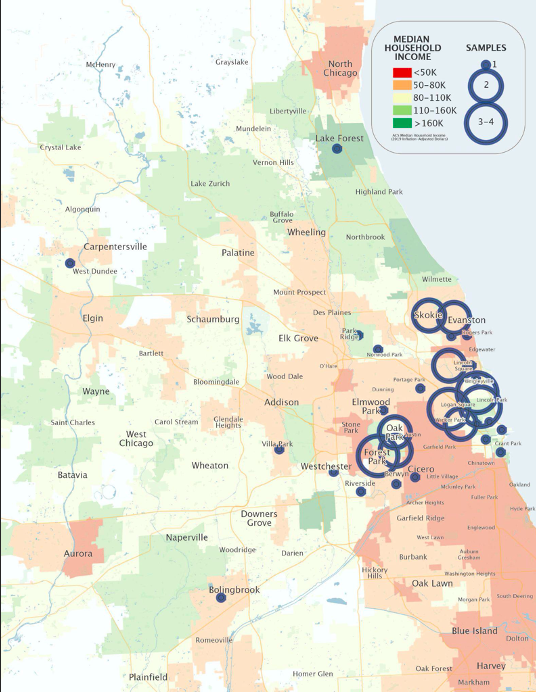

Supplement: Supplementary file 2 — FIGURE S2. Location of study families' residential zip codes across Chicagoland. Blue circles indicate the number of families sampled from a given area, which are labeled by median household income (red = lower; green = higher). This original image was created by Joanna B.B. Simon using publicly available 2019 United States Census data. [file AJHB-37-e23972-s003.png]

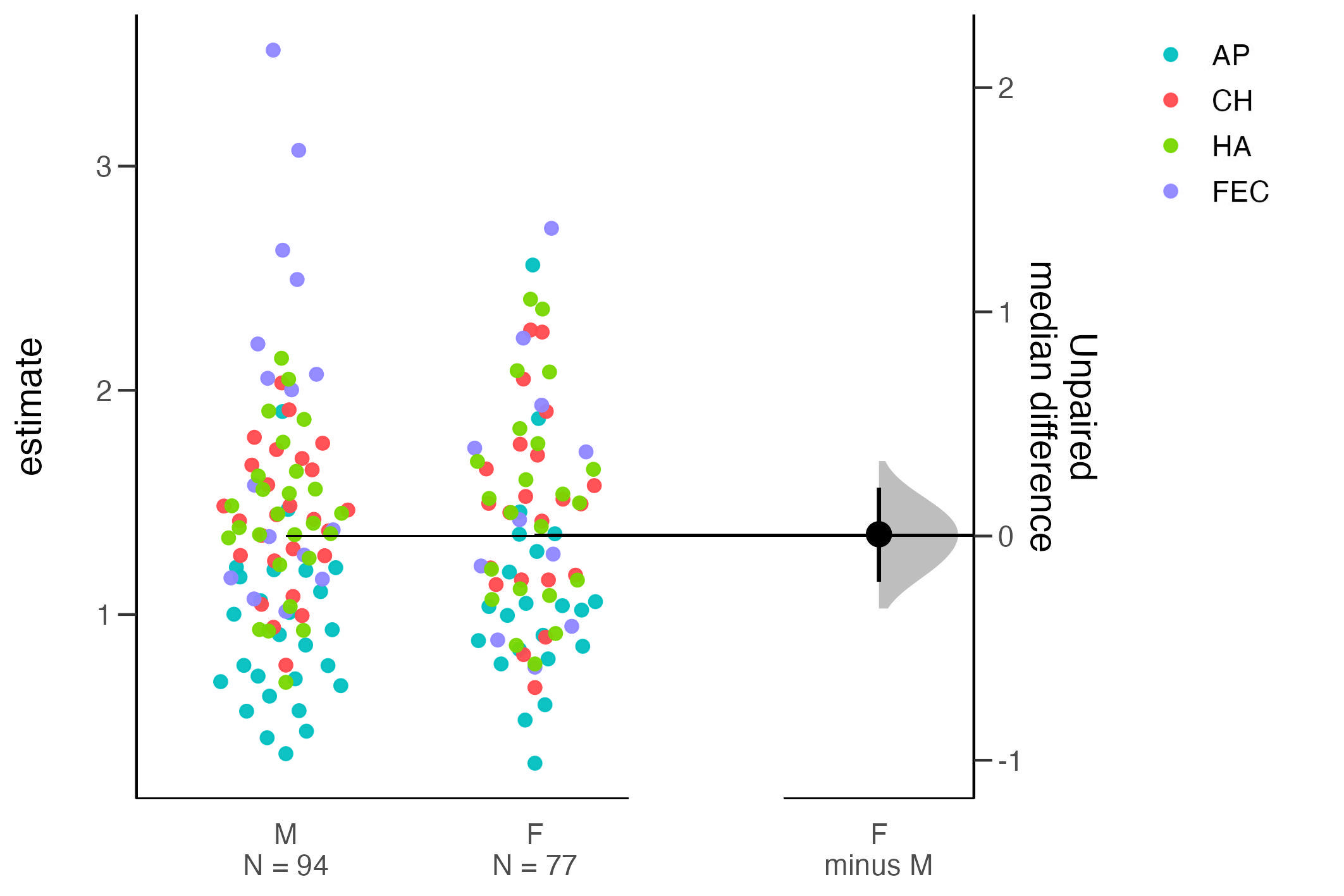

Supplement: Supplementary file 3 — FIGURE S3. Infant bacterial diversity did not show a clear relationship to infant sex. Vertical bars indicate 95% confidence intervals of the median diversity estimate for each group of samples. The bottom panel displays the difference in average diversity between female and male infants. Estimate = estimated Shannon diversity using DivNet; M = male; F = female; AP = axilla; CH = cheek; FEC = fecal; HA = hand. [file AJHB-37-e23972-s008.jpeg]

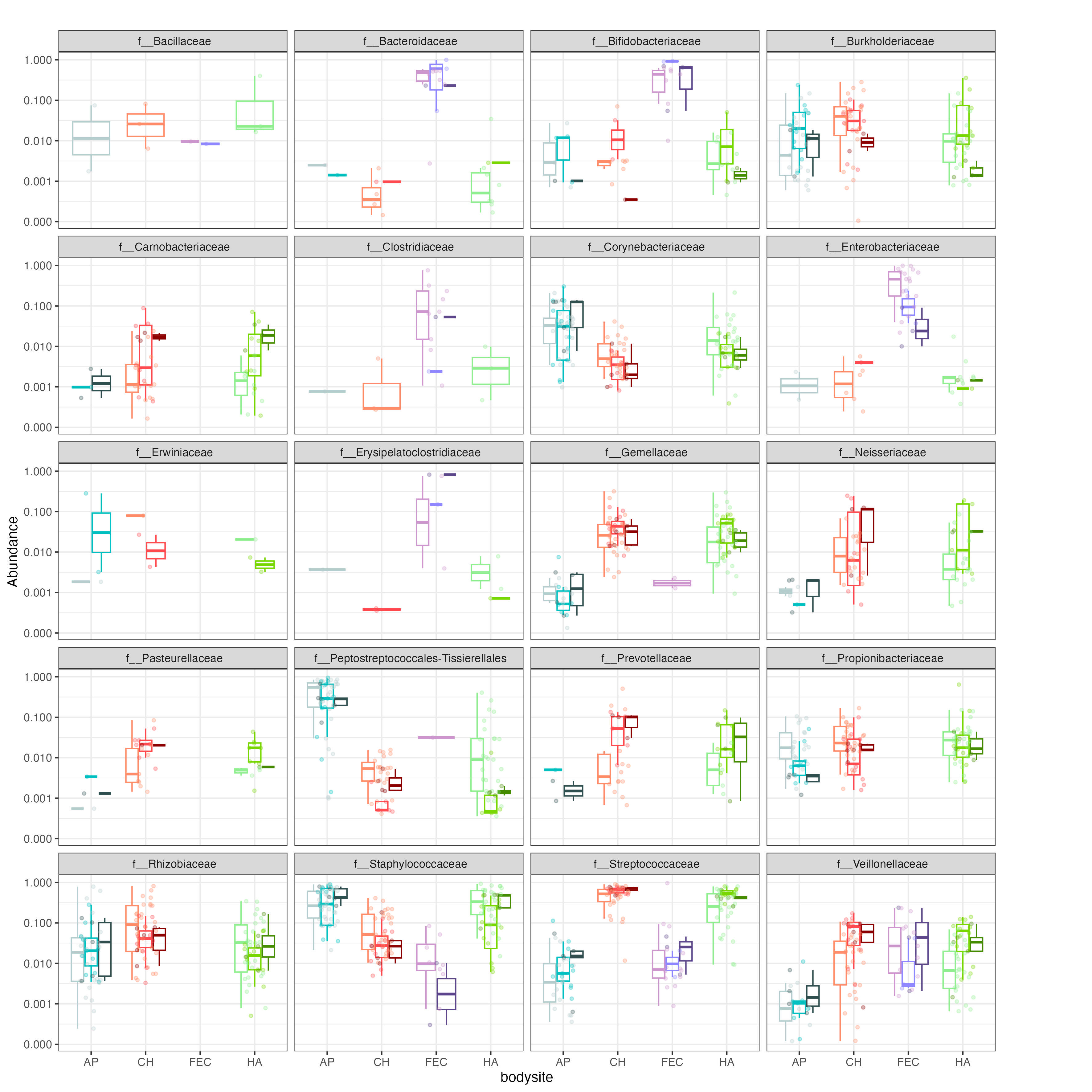

Supplement: Supplementary file 4 — FIGURE S4. The relative abundances of core bacterial families by (a) infant age and (b) body site. In each plot, the x‐axis displays the four infant body sites. Infant age categories are represented by shading (lighter shade = 0–3 months; middle shade = 3–6 months; darker shade = 6+ months). AP = axilla; CH = cheek; FEC = fecal; HA = hand. [file AJHB-37-e23972-s007.zip › FigS4a.age.jpeg]

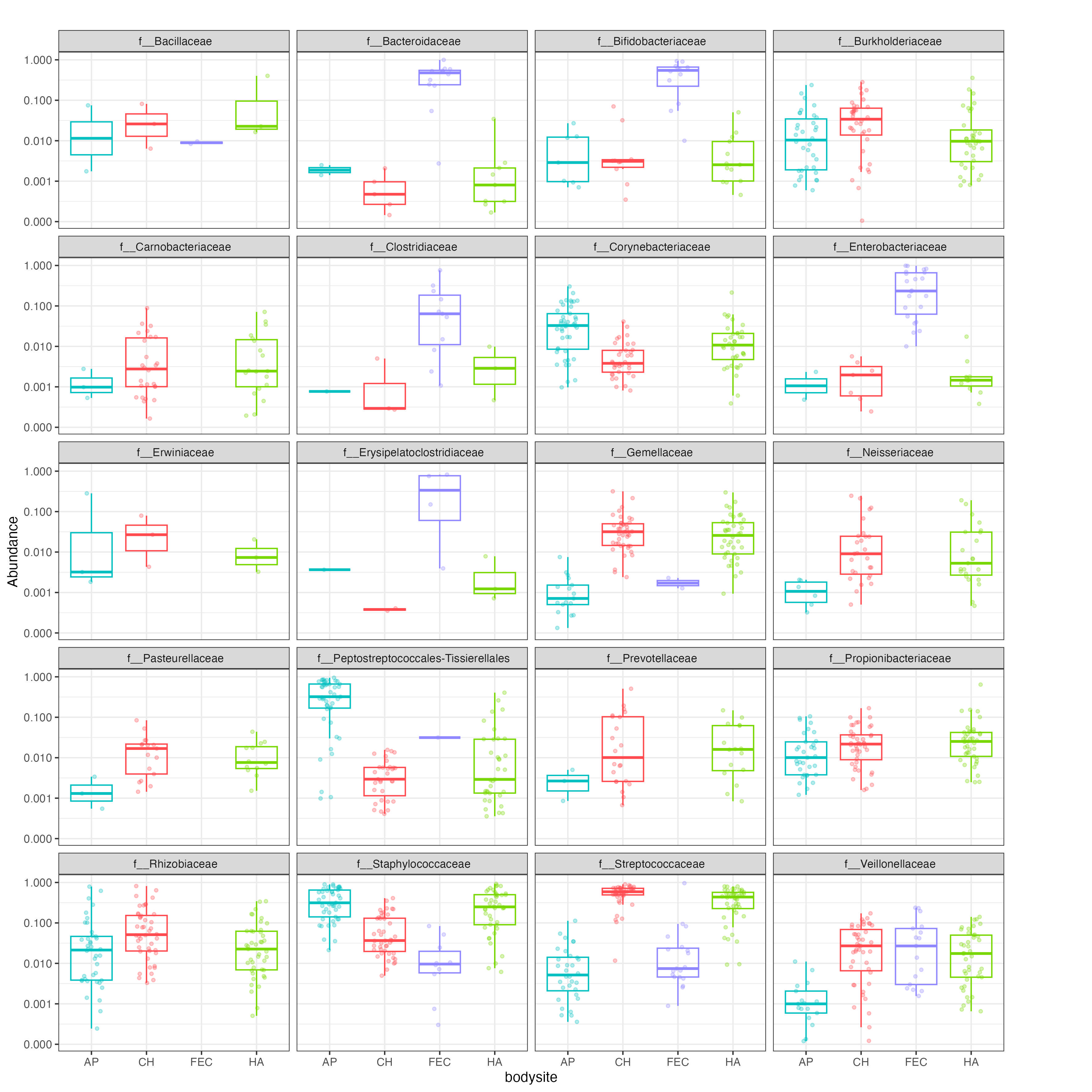

Supplement: Supplementary file 4 — FIGURE S4. The relative abundances of core bacterial families by (a) infant age and (b) body site. In each plot, the x‐axis displays the four infant body sites. Infant age categories are represented by shading (lighter shade = 0–3 months; middle shade = 3–6 months; darker shade = 6+ months). AP = axilla; CH = cheek; FEC = fecal; HA = hand. [file AJHB-37-e23972-s007.zip › FigS4b.bodysite.jpeg]

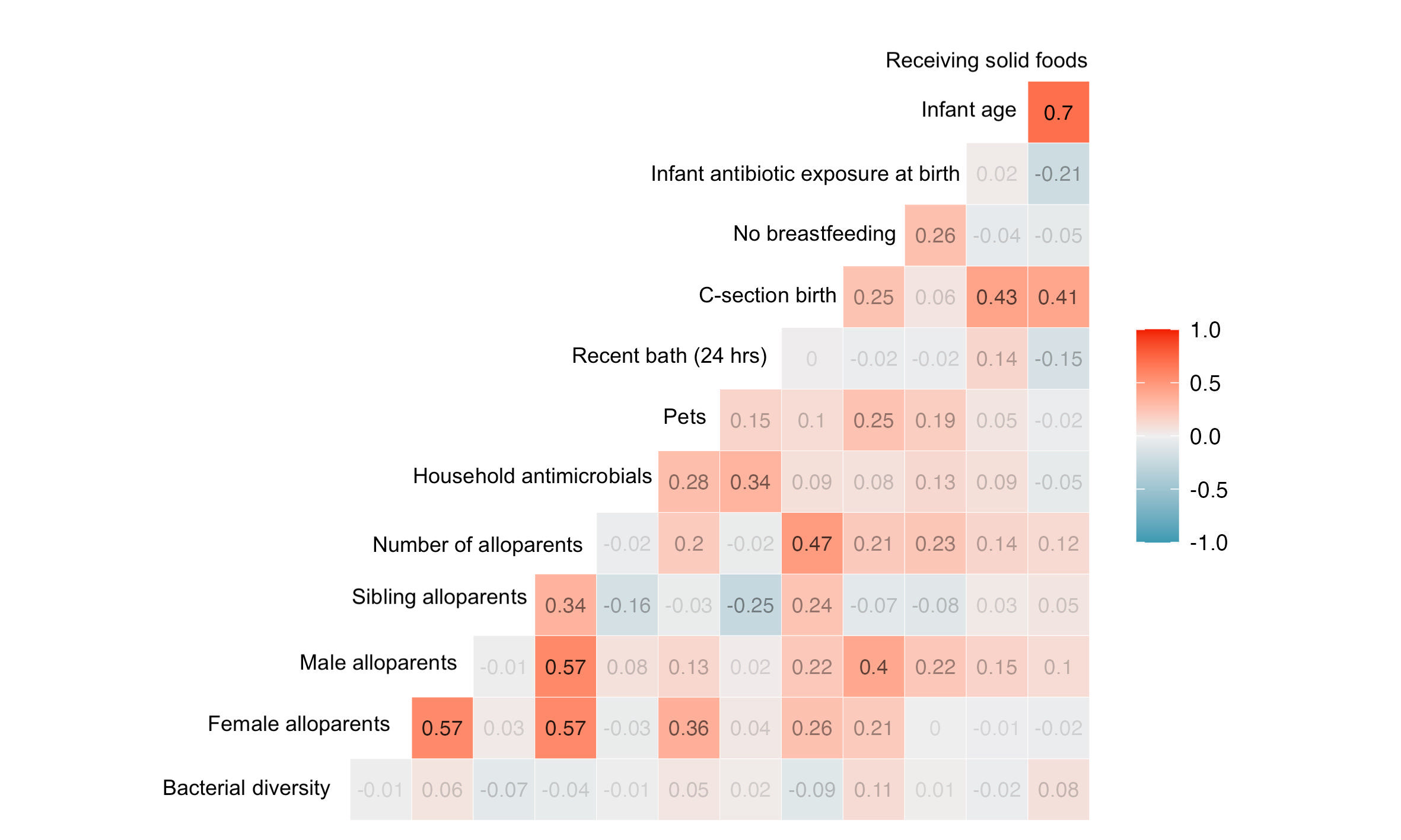

Supplement: Supplementary file 5 — FIGURE S5. Heatmaps illustrating correlations between infant bacterial diversity, environmental variables, and (a) infant‐alloparent relationships or (b) allocare behaviors. [file AJHB-37-e23972-s002.zip › FigS5a.jpeg]

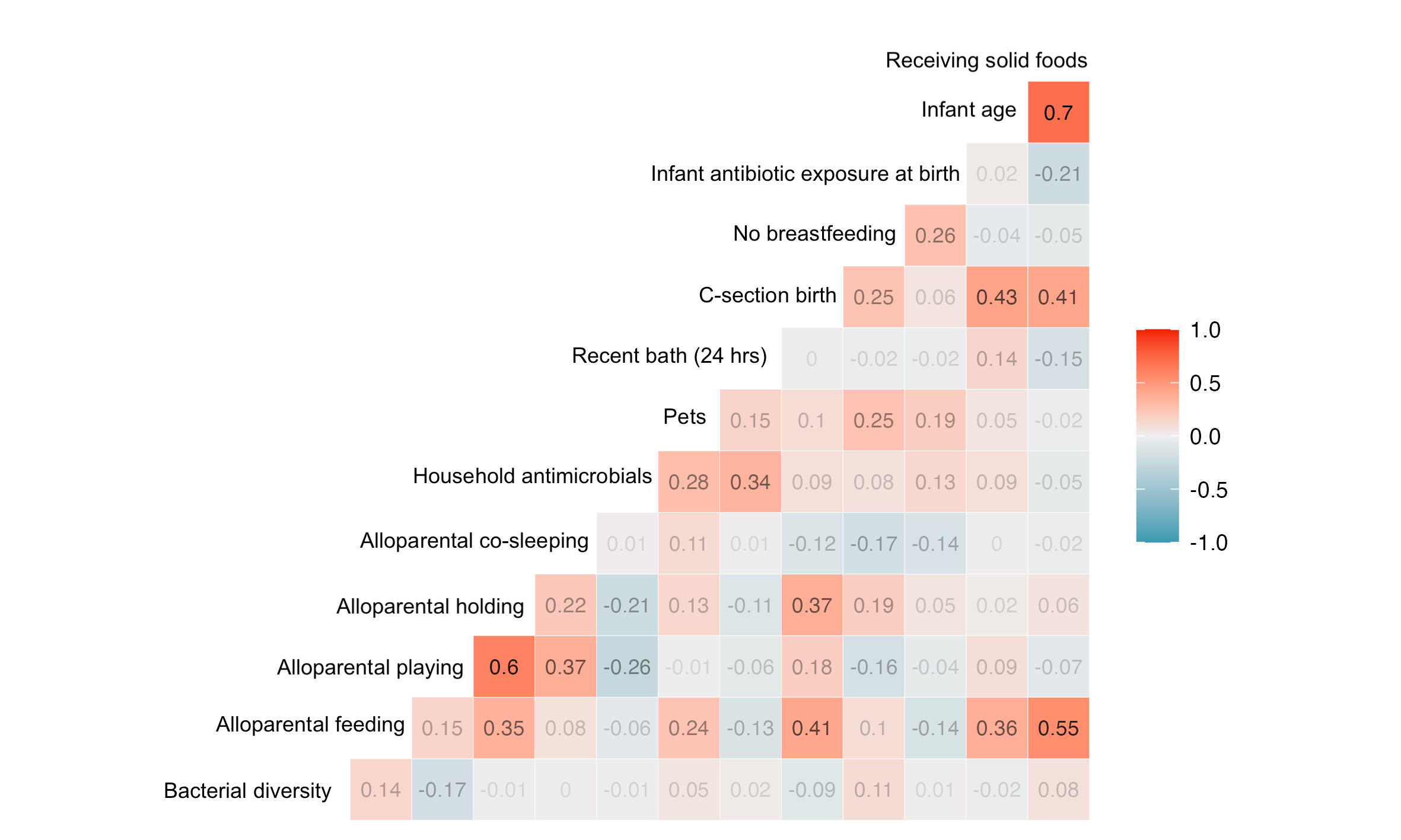

Supplement: Supplementary file 5 — FIGURE S5. Heatmaps illustrating correlations between infant bacterial diversity, environmental variables, and (a) infant‐alloparent relationships or (b) allocare behaviors. [file AJHB-37-e23972-s002.zip › FigS5b.jpeg]
