# Supplementary material for: Interactions with alloparents are associated with the diversity of infant skin and fecal bacterial communities in Chicago, United States
Source: Am J Hum Biol. 2023 Aug 26;37(1):e23972. doi: 10.1002/ajhb.23972 (PMC11667966; doi:10.1002/ajhb.23972)
Supplement: Supplementary file 6 — TABLE S1. Results of regression models displaying associations between infant bacterial diversity and (a) number of alloparents and having (b) female adult alloparents, (c) male adult alloparents, and (d) sibling alloparents. Effect = beta coefficient; SE = standard error; LCI = lower bound of 95% confidence interval; UCI = upper bound of 95% confidence interval. In tables b‐d, models excluded infants with no reported alloparents. CH = cheek; HA = hand; AP = axilla; FEC = fecal. [file AJHB-37-e23972-s005.docx]

1. Number of alloparents

| **CH** | **Effect** | **SE** | **LCI** | **UCI** | **R^2^** |
| --- | --- | --- | --- | --- | --- |
| Number of alloparents | -0.021 | 0.037 | -0.057 | 0.014 | 0.082 |
| Infant age | -0.001 | 0.001 | -0.002 | -0.001 | -- |
| Recent bath | -0.026 | 0.124 | -0.145 | 0.093 | -- |
| C-section birth | -0.069 | 0.191 | -0.252 | 0.115 | -- |
| No breastfeeding | 0.073 | 0.151 | -0.072 | 0.218 | -- |
| Pets | 0.075 | 0.087 | -0.009 | 0.159 |  |
| Intercept | 1.395 | 0.070 | 1.327 | 1.462 | -- |
| **HA** | **Effect** | **SE** | **LCI** | **UCI** | **R^2^** |
| Number of alloparents | 0.035 | 0.044 | -0.007 | 0.077 | 0.096 |
| Infant age | -0.001 | 0.001 | -0.002 | -0.001 | -- |
| Recent bath | 0.249 | 0.141 | 0.114 | 0.384 | -- |
| C-section birth | 0.042 | 0.226 | -0.175 | 0.259 | -- |
| No breastfeeding | -0.075 | 0.164 | -0.233 | 0.083 | -- |
| Pets | -0.145 | 0.099 | -0.239 | -0.050 |  |
| Intercept | 1.429 | 0.081 | 1.352 | 1.507 | -- |
| **AP** | **Effect** | **SE** | **LCI** | **UCI** | **R^2^** |
| Number of alloparents | -0.006 | 0.039 | -0.044 | 0.031 | 0.151 |
| Infant age | -0.002 | 0.001 | -0.003 | -0.001 | -- |
| Recent bath | 0.073 | 0.133 | -0.054 | 0.201 | -- |
| C-section birth | 0.404 | 0.206 | 0.207 | 0.602 | -- |
| No breastfeeding | 0.005 | 0.147 | -0.136 | 0.146 | -- |
| Pets | 0.284 | 0.088 | 0.200 | 0.369 |  |
| Intercept | -0.006 | 0.039 | -0.044 | 0.031 | -- |
| **FEC** | **Effect** | **SE** | **LCI** | **UCI** | **R^2^** |
| Number of alloparents | 0.124 | 0.096 | 0.032 | 0.216 | 0.156 |
| Infant age | 0.001 | 0.001 | 0.001 | 0.002 | -- |
| Recent bath | -0.241 | 0.173 | -0.407 | -0.075 | -- |
| C-section birth | -0.434 | 0.537 | -0.950 | 0.082 | -- |
| Any milk | 0.286 | 0.121 | -0.144 | 0.088 | -- |
| Pets | 0.156 | 0.145 | 0.017 | 0.295 | -- |
| Intercept | 1.523 | 0.114 | 1.414 | 1.632 | -- |

1. Female adult alloparents

| **CH** | **Effect** | **SE** | **LCI** | **UCI** | **R^2^** |
| --- | --- | --- | --- | --- | --- |
| Female alloparents | -0.348 | 0.117 | -0.460 | -0.236 | 0.135 |
| Infant age | -0.001 | 0.001 | -0.002 | 0.000 | -- |
| Recent bath | 0.233 | 0.171 | 0.069 | 0.397 | -- |
| C-section birth | -0.042 | 0.188 | -0.222 | 0.139 | -- |
| No breastfeeding | 0.047 | 0.173 | -0.120 | 0.213 | -- |
| Pets | 0.154 | 0.099 | 0.059 | 0.249 | -- |
| Intercept | 1.434 | 0.084 | 1.353 | 1.515 | -- |
| **HA** | **Effect** | **SE** | **LCI** | **UCI** | **R^2^** |
| Female alloparents | 0.344 | 0.134 | 0.215 | 0.473 | 0.159 |
| Infant age | -0.002 | 0.001 | -0.003 | -0.001 | -- |
| Recent bath | -0.234 | 0.186 | -0.412 | -0.055 | -- |
| C-section birth | 0.145 | 0.222 | -0.068 | 0.358 | -- |
| No breastfeeding | -0.206 | 0.197 | -0.395 | -0.017 | -- |
| Pets | -0.298 | 0.117 | -0.411 | -0.186 | -- |
| Intercept | 1.595 | 0.101 | 1.499 | 1.692 | -- |
| **AP** | **Effect** | **SE** | **LCI** | **UCI** | **R^2^** |
| Female alloparents | 0.229 | 0.108 | 0.125 | 0.334 | 0.285 |
| Infant age | -0.003 | 0.001 | -0.004 | -0.003 | -- |
| Recent bath | 0.188 | 0.160 | 0.035 | 0.341 | -- |
| C-section birth | 0.404 | 0.180 | 0.232 | 0.577 | -- |
| No breastfeeding | -0.237 | 0.157 | -0.388 | -0.086 | -- |
| Pets | 0.372 | 0.092 | 0.283 | 0.460 | -- |
| Intercept | 1.229 | 0.077 | 1.155 | 1.303 | -- |
| **FEC** | **Effect** | **SE** | **LCI** | **UCI** | **R^2^** |
| Female alloparents | 0.331 | 0.175 | 0.162 | 0.499 | 0.421 |
| Infant age | 0.025 | 0.002 | 0.024 | 0.027 | -- |
| Recent bath | -0.748 | 0.219 | -0.958 | -0.538 | -- |
| C-section birth | -2.910 | 0.463 | -3.355 | -2.464 | -- |
| Any milk | -1.306 | 0.149 | -1.450 | -1.163 | -- |
| Pets | 0.360 | 0.155 | 0.211 | 0.509 | -- |
| Intercept | 0.910 | 0.143 | 0.773 | 1.047 | -- |

1. Male adult alloparents

| **CH** | **Effect** | **SE** | **LCI** | **UCI** | **R^2^** |
| --- | --- | --- | --- | --- | --- |
| Male alloparents | -0.117 | 0.205 | -0.314 | 0.080 | 0.077 |
| Infant age | -0.001 | 0.001 | -0.002 | 0.000 | -- |
| Recent bath | 0.109 | 0.179 | -0.063 | 0.281 | -- |
| C-section birth | -0.119 | 0.199 | -0.310 | 0.072 | -- |
| No breastfeeding | 0.000 | 0.183 | -0.175 | 0.176 | -- |
| Pets | 0.011 | 0.105 | -0.090 | 0.111 | -- |
| Intercept | 1.398 | 0.089 | 1.312 | 1.483 | -- |
| **HA** | **Effect** | **SE** | **LCI** | **UCI** | **R^2^** |
| Male alloparents | 0.135 | 0.231 | -0.087 | 0.356 | 0.102 |
| Infant age | -0.002 | 0.001 | -0.003 | -0.001 | -- |
| Recent bath | -0.110 | 0.192 | -0.294 | 0.074 | -- |
| C-section birth | 0.233 | 0.228 | 0.014 | 0.452 | -- |
| No breastfeeding | -0.174 | 0.203 | -0.369 | 0.021 | -- |
| Pets | -0.151 | 0.121 | -0.267 | -0.035 | -- |
| Intercept | 1.636 | 0.104 | 1.536 | 1.736 | -- |
| **AP** | **Effect** | **SE** | **LCI** | **UCI** | **R^2^** |
| Male alloparents | -0.227 | 0.188 | -0.408 | -0.046 | 0.305 |
| Infant age | -0.004 | 0.001 | -0.004 | -0.003 | -- |
| Recent bath | 0.253 | 0.163 | 0.096 | 0.410 | -- |
| C-section birth | 0.456 | 0.184 | 0.279 | 0.633 | -- |
| No breastfeeding | -0.082 | 0.161 | -0.237 | 0.072 | -- |
| Pets | 0.506 | 0.095 | 0.415 | 0.597 | -- |
| Intercept | 1.239 | 0.079 | 1.163 | 1.315 | -- |
| **FEC** | **Effect** | **SE** | **LCI** | **UCI** | **R^2^** |
| Male alloparents | -1.411 | 0.883 | -2.259 | -0.564 | 0.698 |
| Infant age | 0.035 | 0.002 | 0.033 | 0.036 | -- |
| Recent bath | -0.825 | 0.204 | -1.021 | -0.629 | -- |
| C-section birth | -4.129 | 0.449 | -4.560 | -3.698 | -- |
| Any milk | -1.632 | 0.140 | -1.766 | -1.498 | -- |
| Pets | 0.687 | 0.145 | 0.548 | 0.826 | -- |
| Intercept | 0.683 | 0.133 | 0.555 | 0.812 | -- |

1. Sibling alloparents

| **CH** | **Effect** | **SE** | **LCI** | **UCI** | **R^2^** |
| --- | --- | --- | --- | --- | --- |
| Sibling alloparents | -0.088 | 0.114 | -0.198 | 0.021 | 0.076 |
| Infant age | -0.001 | 0.001 | -0.002 | 0.000 | -- |
| Recent bath | 0.083 | 0.179 | -0.089 | 0.255 | -- |
| C-section birth | -0.083 | 0.199 | -0.274 | 0.108 | -- |
| No breastfeeding | -0.075 | 0.183 | -0.251 | 0.101 | -- |
| Pets | -0.021 | 0.105 | -0.122 | 0.079 | -- |
| Intercept | 1.487 | 0.089 | 1.401 | 1.573 | -- |
| **HA** | **Effect** | **SE** | **LCI** | **UCI** | **R^2^** |
| Sibling alloparents | -0.040 | 0.142 | -0.176 | 0.097 | 0.088 |
| Infant age | -0.002 | 0.001 | -0.003 | -0.001 | -- |
| Recent bath | -0.112 | 0.192 | -0.296 | 0.072 | -- |
| C-section birth | 0.233 | 0.228 | 0.014 | 0.452 | -- |
| No breastfeeding | -0.125 | 0.203 | -0.320 | 0.070 | -- |
| Pets | -0.159 | 0.121 | -0.275 | -0.043 | -- |
| Intercept | 1.667 | 0.104 | 1.567 | 1.767 | -- |
| **AP** | **Effect** | **SE** | **LCI** | **UCI** | **R^2^** |
| Sibling alloparents | -0.356 | 0.093 | -0.445 | -0.266 | 0.402 |
| Infant age | -0.004 | 0.001 | -0.004 | -0.003 | -- |
| Recent bath | 0.183 | 0.157 | 0.033 | 0.334 | -- |
| C-section birth | 0.565 | 0.176 | 0.395 | 0.734 | -- |
| No breastfeeding | -0.287 | 0.154 | -0.434 | -0.139 | -- |
| Pets | 0.358 | 0.090 | 0.272 | 0.445 | -- |
| Intercept | 1.595 | 0.075 | 1.523 | 1.666 | -- |
| **FEC** | **Effect** | **SE** | **LCI** | **UCI** | **R^2^** |
| Sibling alloparents | 0.435 | 0.194 | 0.249 | 0.621 | 0.671 |
| Infant age | 0.022 | 0.002 | 0020 | 0.023 | -- |
| Recent bath | -0.544 | 0.209 | -0.744 | -0.344 | -- |
| C-section birth | -2.385 | 0.454 | -2.821 | -1.949 | -- |
| Any milk | -1.356 | 0.143 | -1.493 | -1.212 | -- |
| Pets | 0.882 | 0.148 | 0.739 | 1.024 | -- |
| Intercept | 0.620 | 0.137 | 0.489 | 0.751 | -- |
