# Supplementary material for: Interactions with alloparents are associated with the diversity of infant skin and fecal bacterial communities in Chicago, United States
Source: Am J Hum Biol. 2023 Aug 26;37(1):e23972. doi: 10.1002/ajhb.23972 (PMC11667966; doi:10.1002/ajhb.23972)
Supplement: Supplementary file 7 — TABLE S2. Results of regression models displaying associations between infant bacterial diversity and alloparental (a) feeding, (b) co‐sleeping, (c) holding, and (d) playing. Effect = beta coefficient; SE = standard error; LCI = lower bound of 95% confidence interval; UCI = upper bound of 95% confidence interval. CH = cheek; HA = hand; AP = axilla; FEC = fecal. [file AJHB-37-e23972-s004.docx]

1. Feeding

| CH | Effect | SE | LCI | UCI | R^2 |
| --- | --- | --- | --- | --- | --- |
| Alloparent feed | -0.323 | 0.197 | -0.511 | -0.134 | 0.201 |
| Father feed | -0.079 | 0.112 | -0.187 | 0.029 | -- |
| Infant age | 0.000 | 0.001 | -0.001 | 0.001 | -- |
| Recent bath | 0.008 | 0.181 | -0.166 | 0.182 | -- |
| C-section birth | -0.093 | 0.201 | -0.287 | 0.100 | -- |
| No breastfeeding | -0.018 | 0.185 | -0.196 | 0.160 | -- |
| Pets | 0.149 | 0.106 | 0.047 | 0.252 | -- |
| Intercept | 1.330 | 0.090 | 1.243 | 1.417 | -- |

| HA | Effect | SE | LCI | UCI | R^2 |
| --- | --- | --- | --- | --- | --- |
| Alloparent feed | -0.602 | 0.222 | -0.815 | -0.389 | 0.237 |
| Father feed | 0.393 | 0.118 | 0.280 | 0.507 | -- |
| Infant age | -0.001 | 0.001 | -0.002 | 0.000 | -- |
| Recent bath | -0.244 | 0.174 | -0.411 | -0.077 | -- |
| C-section birth | 0.300 | 0.209 | 0.099 | 0.501 | -- |
| No breastfeeding | -0.289 | 0.185 | -0.467 | -0.112 | -- |
| Pets | -0.088 | 0.110 | -0.193 | 0.018 | -- |
| Intercept | 1.389 | 0.095 | 1.298 | 1.479 | -- |

| AP | Effect | SE | LCI | UCI | R^2 |
| --- | --- | --- | --- | --- | --- |
| Alloparent feed | -0.015 | 0.199 | -0.206 | 0.176 | 0.339 |
| Father feed | 0.148 | 0.104 | 0.048 | 0.248 | -- |
| Infant age | -0.003 | 0.001 | -0.004 | -0.003 | -- |
| Recent bath | 0.255 | 0.174 | 0.088 | 0.421 | -- |
| C-section birth | 0.456 | 0.196 | 0.268 | 0.645 | -- |
| No breastfeeding | -0.231 | 0.173 | -0.397 | -0.065 | -- |
| Pets | 0.442 | 0.102 | 0.344 | 0.540 | -- |
| Intercept | 1.173 | 0.085 | 1.091 | 1.255 | -- |

1. Co-sleeping

| CH | Effect | SE | LCI | UCI | R^2 |
| --- | --- | --- | --- | --- | --- |
| Alloparent sleep | -0.163 | 0.201 | -0.356 | 0.031 | 0.095 |
| Father sleep | -0.324 | 0.108 | -0.428 | -0.221 | -- |
| Infant age | -0.001 | 0.001 | -0.002 | 0.000 | -- |
| Recent bath | 0.105 | 0.172 | -0.061 | 0.270 | -- |
| C-section birth | -0.312 | 0.190 | -0.494 | -0.129 | -- |
| No breastfeeding | -0.265 | 0.175 | -0.433 | -0.096 | -- |
| Pets | 0.014 | 0.100 | -0.082 | 0.110 | -- |
| Intercept | 1.744 | 0.085 | 1.663 | 1.826 | -- |

| HA | Effect | SE | LCI | UCI | R^2 |
| --- | --- | --- | --- | --- | --- |
| Alloparent sleep | 0.368 | 0.269 | 0.110 | 0.626 | 0.158 |
| Father sleep | 0.184 | 0.131 | 0.058 | 0.310 | -- |
| Infant age | -0.002 | 0.001 | -0.003 | -0.001 | -- |
| Recent bath | -0.097 | 0.184 | -0.274 | 0.080 | -- |
| C-section birth | 0.382 | 0.220 | 0.170 | 0.593 | -- |
| No breastfeeding | 0.033 | 0.195 | -0.155 | 0.220 | -- |
| Pets | -0.156 | 0.116 | -0.268 | -0.045 | -- |
| Intercept | 1.416 | 0.100 | 1.320 | 1.512 | -- |

| AP | Effect | SE | LCI | UCI | R^2 |
| --- | --- | --- | --- | --- | --- |
| Alloparent sleep | 0.028 | 0.217 | -0.180 | 0.236 | 0.314 |
| Father sleep | -0.309 | 0.099 | -0.404 | -0.214 | -- |
| Infant age | -0.004 | 0.001 | -0.004 | -0.003 | -- |
| Recent bath | 0.211 | 0.165 | 0.053 | 0.370 | -- |
| C-section birth | 0.279 | 0.186 | 0.100 | 0.458 | -- |
| No breastfeeding | -0.328 | 0.163 | -0.484 | -0.171 | -- |
| Pets | 0.488 | 0.096 | 0.396 | 0.581 | -- |
| Intercept | 1.528 | 0.080 | 1.451 | 1.605 | -- |

| FEC | Effect | SE | LCI | UCI | R^2 |
| --- | --- | --- | --- | --- | --- |
| Alloparent sleep | 0.677 | 0.148 | 0.535 | 0.819 | 0.904 |
| Father sleep | 0.319 | 0.109 | 0.214 | 0.424 | -- |
| Infant age | 0.020 | 0.001 | 0.019 | 0.021 | -- |
| Recent bath | -0.206 | 0.123 | -0.324 | -0.087 | -- |
| C-section birth | -2.076 | 0.384 | -2.444 | -1.708 | -- |
| Any milk | -1.322 | 0.086 | -1.405 | -1.239 | -- |
| Pets | 0.104 | 0.088 | 0.020 | 0.188 | -- |
| Intercept | 1.1 04 | 0.084 | 1.021 | 1.182 | -- |

1. Holding

| CH | Effect | SE | LCI | UCI | R^2 |
| --- | --- | --- | --- | --- | --- |
| Alloparent hold | -0.124 | 0.097 | -0.217 | -0.031 | 0.082 |
| Infant age | -0.001 | 0.001 | -0.002 | 0.000 | -- |
| Recent bath | 0.089 | 0.180 | -0.084 | 0.262 | -- |
| C-section birth | -0.082 | 0.200 | -0.274 | 0.109 | -- |
| No breastfeeding | -0.033 | 0.184 | -0.209 | 0.143 | -- |
| Pets | -0.003 | 0.105 | -0.105 | 0.098 | -- |
| Intercept | 1.530 | 0.089 | 1.444 | 1.616 | -- |

| HA | Effect | SE | LCI | UCI | R^2 |
| --- | --- | --- | --- | --- | --- |
| Alloparent hold | 0.001 | 0.112 | -0.106 | 0.109 | 0.086 |
| Infant age | -0.002 | 0.001 | -0.003 | -0.001 | -- |
| Recent bath | -0.103 | 0.192 | -0.287 | 0.082 | -- |
| C-section birth | 0.220 | 0.229 | 0.000 | 0.439 | -- |
| No breastfeeding | -0.115 | 0.203 | -0.310 | 0.081 | -- |
| Pets | -0.145 | 0.121 | -0.261 | -0.028 | -- |
| Intercept | 1.628 | 0.104 | 1.528 | 1.728 | -- |

| AP | Effect | SE | LCI | UCI | R^2 |
| --- | --- | --- | --- | --- | --- |
| Alloparent hold | 0.315 | 0.083 | 0.235 | 0.395 | 0.307 |
| Infant age | -0.003 | 0.001 | -0.004 | -0.002 | -- |
| Recent bath | 0.261 | 0.160 | 0.107 | 0.414 | -- |
| C-section birth | 0.359 | 0.180 | 0.186 | 0.532 | -- |
| No breastfeeding | -0.228 | 0.157 | -0.379 | -0.077 | -- |
| Pets | 0.546 | 0.092 | 0.457 | 0.634 | -- |
| Intercept | 0.908 | 0.077 | 0.834 | 0.982 | -- |

| FEC | Effect | SE | LCI | UCI | R^2 |
| --- | --- | --- | --- | --- | --- |
| Alloparent hold | 0.146 | 0.163 | -0.010 | 0.302 | 0.403 |
| Infant age | 0.025 | 0.002 | 0.023 | 0.026 | -- |
| Recent bath | -0.689 | 0.237 | -0.917 | -0.462 | -- |
| C-section birth | -2.825 | 0.484 | -3.289 | -2.360 | -- |
| Any milk | -1. 378 | 0.163 | -0.010 | 0.302 | -- |
| Pets | 0.639 | 0.168 | 0.477 | 0.800 | -- |
| Intercept | 0.826 | 0.154 | 0.678 | 0.974 | -- |

1. Playing

| CH | Effect | SE | LCI | UCI | R^2 |
| --- | --- | --- | --- | --- | --- |
| Alloparent play | 0.224 | 0.097 | 0.130 | 0.317 | 0.187 |
| Father play | 0.511 | 0.090 | 0.424 | 0.597 | -- |
| Infant age | -0.001 | 0.001 | -0.001 | 0.000 | -- |
| Recent bath | 0.014 | 0.176 | -0.155 | 0.183 | -- |
| C-section birth | 0.011 | 0.195 | -0.175 | 0.198 | -- |
| No breastfeeding | 0.164 | 0.179 | -0.008 | 0.336 | -- |
| Pets | 0.092 | 0.103 | -0.006 | 0.191 | -- |
| Intercept | 0.604 | 0.087 | 0.521 | 0.688 | -- |

| HA | Effect | SE | LCI | UCI | R^2 |
| --- | --- | --- | --- | --- | --- |
| Alloparent play | -0.135 | 0.114 | -0.245 | -0.026 | 0.161 |
| Father play | 0.920 | 0.103 | 0.821 | 1.018 | -- |
| Infant age | -0.002 | 0.001 | -0.003 | -0.001 | -- |
| Recent bath | -0.237 | 0.183 | -0.413 | -0.061 | -- |
| C-section birth | 0.451 | 0.219 | 0.241 | 0.661 | -- |
| No breastfeeding | -0.017 | 0.194 | -0.204 | 0.169 | -- |
| Pets | -0.114 | 0.116 | -0.225 | -0.003 | -- |
| Intercept | 0.779 | 0.099 | 0.683 | 0.874 | -- |

| AP | Effect | SE | LCI | UCI | R^2 |
| --- | --- | --- | --- | --- | --- |
| Alloparent play | -0.042 | 0.091 | -0.130 | 0.045 | 0.331 |
| Father play | 0.490 | 0.085 | 0.409 | 0.571 | -- |
| Infant age | -0.003 | 0.001 | -0.004 | -0.003 | -- |
| Recent bath | 0.178 | 0.169 | 0.016 | 0.340 | -- |
| C-section birth | 0.588 | 0.190 | 0.406 | 0.771 | -- |
| No breastfeeding | -0.105 | 0.167 | -0.265 | 0.055 | -- |
| Pets | 0.508 | 0.098 | 0.413 | 0.602 | -- |
| Intercept | 0.753 | 0.082 | 0.674 | 0.832 | -- |
